# Anisotropic Behavior of 3D-Printed Concrete: Interlayer Bonding, Pore Architecture, Reinforcement Limitations, and Durability Mechanisms

**DOI:** 10.3390/ma19173698

**Published:** 2026-08-31

**Authors:** Ali Mardani, Mohammad Hematibahar, Selin Özteber, Qais Abdulrahman Ali Qais, Ivan Khalil, Tesfaldet Hadgembes Gebre, Ahmed Elsheikh

**Affiliations:** 1 Department of Civil Engineering, Bursa Uludag University, Bursa 16059, Türkiye; ozteberselin@uludag.edu.tr; 2 Department of Architecture, Restoration and Design, Peoples’ Friendship University of Russia (RUDN), 117198 Moscow, Russia; eng.m.hematibahar1994@gmail.com (M.H.); tesfaldethg@gmail.com (T.H.G.); 3 Department of Construction Technologies and Structural Materials, Peoples’ Friendship University of Russia (RUDN), 117198 Moscow, Russia; qaiseng@gmail.com; 4 Department of Industrial and Architectural Design, Peoples’ Friendship University of Russia (RUDN), 117198 Moscow, Russia; khalil-i@rudn.ru; 5 College of Aerospace and Civil Engineering, Harbin Engineering University, Harbin 150001, China; ahmed.elshiekh19@feng.bu.edu.eg

**Keywords:** 3D printed concrete, anisotropy, interlayer bonding, cold joint, mechanical interlocking, fiber reinforcement, durability, structural performance

## Abstract

The structural use of three-dimensional concrete printing remains limited by the directional weakness introduced during extrusion and layer-by-layer deposition. Although 3DPC offers major advantages in formwork elimination, architectural freedom, and automated construction, its printed architecture produces interfaces, pore networks, and reinforcement discontinuities that do not exist in the same form in conventionally cast concrete. This review examines the anisotropic behavior of 3DPC by linking its architectural arrangement, physical interlayer mechanisms, and chemical durability-related processes. The analysis shows that anisotropy develops from the combined effects of filament orientation, interlayer bonding quality, pore morphology, cold-joint formation, mechanical interlocking, hydration continuity, and reinforcement limitations. Weak interlayer regions act not only as preferred paths for crack initiation and propagation under tensile, flexural, shear, and compressive loading, but also as transport channels that accelerate water absorption, chloride ingress, carbonation, sulfate attack, and freeze–thaw deterioration. The review further highlights that fiber, textile, FRP, and discrete reinforcement strategies can reduce some consequences of anisotropy, but their effectiveness depends on whether they bridge the weaker interlayer regions rather than merely reinforcing the filament direction. SEM-based observations confirm that microstructural discontinuities, fiber-matrix debonding, irregular hydration products, and connected pores provide the material-level basis for the directional response of printed concrete. Overall, anisotropy should be treated as a design-critical feature of 3DPC rather than as a secondary defect. Reliable structural application requires coordinated control of mixture rheology, deposition parameters, interlayer timing, curing, toolpath design, and reinforcement layout.

## 1. Introduction

Three-dimensional concrete printing (3DPC) has become one of the most significant developments in digital construction because it reduces the dependence on formwork, allows automated material deposition, and enables geometries that are difficult to achieve with conventional casting [1,2,3,4]. In extrusion-based 3DPC, a cementitious mixture is deposited layer by layer along a predefined toolpath, and the final shape is produced through the controlled movement of the printing system rather than by casting into a mould. This approach offers clear advantages in terms of architectural freedom, material-efficient design, and reduction in construction waste, particularly when the printed geometry is adapted to the expected load path and functional requirements [5,6,7]. Nevertheless, these advantages are accompanied by mechanical and durability-related limitations that are not encountered in the same form in conventionally cast concrete. Printed elements often show weaker interlayer regions, higher sensitivity to processing parameters, and a more pronounced dependence of strength on loading direction [8,9,10].

The performance of 3DPC is controlled by the interaction between mixture design, fresh-state rheology, printing parameters, and the geometry of the printed element. Pumpability, extrudability, buildability, open time, thixotropic structural build-up, nozzle geometry, deposition rate, and interlayer time govern whether the material can be extruded continuously, retain its shape after deposition, and bond adequately with the previously printed layer [11,12,13]. Unlike cast concrete, which is generally compacted within formwork by vibration or designed to consolidate under its own weight, 3DPC is placed without external consolidation. Consequently, entrapped air, non-uniform pore distribution, imperfect layer contact, and local discontinuities may remain within or between the deposited filaments. These features should not be regarded only as printing defects; they directly influence stress transfer, crack initiation, permeability paths, and the direction-dependent mechanical response of the printed body.

Reinforcement is another unresolved issue that restricts the structural use of 3DPC. Several studies have examined printable geopolymer or cementitious materials without conventional reinforcement, while recent demonstrations have shown that unreinforced printed forms, such as masonry-type arches, can be structurally feasible when the geometry is carefully designed [14,15]. However, the absence of continuous reinforcement remains a critical limitation for elements governed by bending, tensile stresses, ductility, crack control, and long-term serviceability. This limitation becomes more severe because the printed material itself contains preferential planes of weakness. Interlayer bond strength is generally lower than the strength of the bulk filament, and the quality of the interface is strongly affected by surface moisture loss, setting progress, filament geometry, deposition interval, and air entrapment during printing [16,17,18,19]. Therefore, the mechanical performance of 3DPC cannot be assessed only from the compressive strength of the printable mixture; the continuity and quality of the printed interfaces must also be considered.

Anisotropy is therefore not a secondary imperfection of 3DPC but a direct consequence of the extrusion and layer deposition process. The printed element behaves differently depending on the orientation of the applied load relative to the filament direction and the interlayer interface. Under compression, flexure, tension, and shear, failure may occur through the filament core, along the interlayer region, or through a combined path crossing both zones [20,21,22,23]. This directional response is produced by several coupled mechanisms: preferential alignment of particles and fibers during extrusion, non-uniform compaction across the filament section, moisture exchange at the exposed layer surface, incomplete mechanical interlocking between consecutive layers, and the development of pores or cold joints at the interface. For this reason, anisotropy should not be interpreted only through strength ratios measured in different directions. It should be understood as a printing-induced material architecture in which the filament core, filament surface, and interlayer zone have different microstructural and mechanical characteristics.

The influence of fibers further illustrates the complexity of anisotropic behavior in 3DPC. Fiber addition can improve tensile resistance, flexural capacity, crack bridging, and post-cracking performance of cementitious materials [24,25,26]. However, in extrusion-based printing, fibers are not randomly distributed in the same manner as in cast concrete. They tend to align along the direction of material flow, and this alignment may improve the response in one direction while leaving the transverse or interlayer direction insufficiently reinforced [27,28]. The effectiveness of fiber reinforcement therefore depends not only on fiber type and volume fraction, but also on aspect ratio, geometry, dispersion, orientation, and the interaction between the fiber and the fresh matrix during extrusion. Recent studies on fiber-reinforced printable cementitious systems have shown that fiber content and aspect ratio affect both fresh-state stability and hardened mechanical performance, while studies on printed fiber geometry have indicated that fracture behavior is sensitive to the spatial arrangement of reinforcement within the printed body [19,29,30]. These findings indicate that fiber reinforcement should not be treated as a simple solution to anisotropy; it is beneficial only when it improves stress transfer across weak regions rather than merely reinforcing the already stronger extrusion direction.

The interlayer region is the most critical zone for understanding anisotropic weakness. During the time interval between two consecutive layers, the surface of the previously deposited filament undergoes moisture loss, early structural build-up, and partial loss of plasticity. If the next layer is deposited after the surface has become too stiff or too dry, intimate contact between layers is reduced and the interface may behave as a weak plane. This condition promotes interlayer debonding, crack propagation along the layer boundary, and increased permeability. The rheological evolution of the material is therefore directly linked to anisotropy. A rapid increase in yield stress may improve buildability and reduce deformation during printing, but it can also reduce interlayer adhesion if the fresh material can no longer merge with the previous layer. Conversely, insufficient structural build-up may preserve interlayer contact but lead to excessive deformation, loss of dimensional stability, or collapse. The design of 3DPC mixtures must therefore balance shape retention with interlayer continuity, rather than optimizing buildability alone [11,12,13,31].

The chemical and microstructural development of the printed interface also contributes to anisotropic behavior. Hydration products such as calcium silicate hydrate and calcium hydroxide form within a matrix that has already been shaped by extrusion, surface exposure, and interlayer contact conditions. When surface drying, delayed deposition, or poor layer contact interrupts hydration across the interface, the resulting interlayer zone may contain higher porosity, lower continuity of hydration products, and weaker mechanical bonding than the filament core. SEM- and EDS-based studies on cementitious materials confirm that the formation and distribution of calcium silicate hydrate are closely related to packing density, pore refinement, and mechanical performance [32]. In 3DPC, this relationship becomes particularly important because the interface is exposed before the next layer is placed. As a result, the hydration products do not necessarily develop with the same continuity across the layer boundary as they do within the bulk filament. This explains why interlayer defects, pore morphology, and hydration discontinuity are repeatedly associated with reduced bond strength, lower durability, and direction-dependent mechanical response.

Although many studies have examined printability, rheology, buildability, interlayer adhesion, reinforcement, and durability of 3DPC, these topics are still often discussed as separate material or process issues. Recent reviews have advanced specific aspects of this field: Ding et al. [33] provided a critical synthesis of the microstructure and mechanical properties of the interlayer region, while Baktheer and Classen [34] reviewed numerical modeling strategies for the anisotropic behavior of hardened 3DPC, organizing existing approaches into macro-scale phenomenological, macro-scale interface-based, and meso-scale discrete model categories. These contributions remain focused on a single dimension of the problem—microstructural characterization in the first case, computational representation in the second—and, among the reviews compared in Table 1, neither appears to jointly address how architectural arrangement, interfacial physics, and chemical durability mechanisms determine the directional response of printed concrete. Table 1 summarizes the scope of these reviews relative to the present work. The central motivation of this review is that anisotropy cannot be fully explained by any one of these factors alone. It emerges from their combined effect during printing: the architectural arrangement of filaments defines the macroscopic load path; the physical condition of the interface controls stress transfer and crack propagation; and the chemical development of hydration products determines the continuity and durability of the bonded region. Therefore, a more useful interpretation of anisotropy requires linking geometry, interface morphology, pore structure, hydration continuity, fiber orientation, and reinforcement limitations within the same framework.

This review examines the anisotropic behavior of 3DPC from architectural, physical, and chemical perspectives. It first discusses how layer arrangement, toolpath design, filament geometry, and pore architecture affect the directional response of printed elements. It then evaluates the role of cold joints, mechanical interlocking, reinforcement limitations, fiber orientation, and interlayer bonding in the development of anisotropic weakness. Finally, it considers the chemical and durability-related mechanisms associated with hydration disruption, carbonation, sulfate attack, chloride ingress, and freeze–thaw exposure. By connecting these mechanisms, the review aims to clarify why anisotropy remains one of the main barriers to the reliable structural use of 3DPC and how mixture design, printing parameters, reinforcement strategy, and interfacial control can be directed toward more robust printed concrete elements.

The sections that follow are organized around the causal pathway from printing process to interlayer/pore architecture, mechanical and durability response, and reinforcement mitigation, as outlined in Table 2.

## 2. Printed Architecture and Structural Anisotropy

### 2.1. Hierarchical Anisotropy

The anisotropy of 3D-printed concrete is not governed by a single interface or by one printing parameter alone. It develops hierarchically, beginning at the scale of the extruded filament and extending to the layer, interface, toolpath, and structural levels. At the filament scale, the extrusion process induces non-uniform compaction, particle rearrangement, and preferential orientation of elongated constituents along the printing direction. At the layer scale, the contact between successive filaments creates interlayer regions whose mechanical properties differ from those of the filament core. At the structural scale, the global response depends on how these layers and interfaces are arranged relative to the applied load. Therefore, the anisotropic behavior of 3DPC should be interpreted as a consequence of the printed material architecture rather than as a simple directional variation in strength.

Layer deposition strategy, nozzle height, nozzle speed, printing direction, and interlayer time are among the most influential parameters controlling the mechanical response of 3DPC [35,36,37]. These parameters affect not only the external geometry of the printed layer but also the density, pore distribution, surface quality, and degree of contact between adjacent filaments. A nozzle placed too high may produce insufficient compression and poor contact with the previous layer, whereas an excessively low nozzle height may disturb the deposited filament, cause lateral spreading, or create local defects. Similarly, deposition speed influences the degree of filament continuity, the time available for interfacial contact, and the stability of the printed path. These effects become particularly important because the material is not vibrated or mechanically compacted after extrusion.

The layer-wise nature of extrusion gives 3DPC a laminated character, in which the interlayer zone often becomes the preferred path for damage initiation and propagation [4,38,39]. When the applied stress is parallel to the printing direction, the load is mainly carried through the continuous filament body. In contrast, when the load acts perpendicular or inclined to the layer interface, the response becomes more dependent on interlayer bond quality, mechanical interlocking, and the continuity of hydration products across the interface. This explains why specimens printed with the same mixture can exhibit different compressive, tensile, or flexural strengths depending on the loading direction and the orientation of the printed layers [20,21,22,23,40,41,42]. Under flexure, interlayer separation is especially critical because tensile stresses tend to concentrate along the weaker layer boundary, allowing cracks to initiate or propagate through the interface rather than through the stronger filament core [21,40]. Table 3 summarizes representative anisotropy ratios reported in the literature, illustrating how these ratios vary with mixture composition, printing geometry, and interlayer time gap.

The distinction between interlayer, intralayer, and inter-strip regions is essential for understanding hierarchical anisotropy. The interlayer region refers to the horizontal interface between two successive deposited layers, whereas the inter-strip region forms between adjacent filaments placed side by side within the same layer. These two interfaces do not necessarily have the same contact history, compaction state, moisture condition, or pore morphology. As a result, their contribution to failure may differ under compression, shear, and flexure. In compression, the final response may involve a combined contribution from the filament core, interlayer interfaces, and inter-strip contacts. In flexure or tension, however, the weakest interface tends to dominate the crack path. Treating 3DPC as a homogeneous material can therefore obscure the actual failure mechanism.

Rheology and geometric stability are closely connected to this hierarchical response. A printable mixture must possess sufficient yield stress and structural build-up to maintain the deposited shape, but it must also preserve enough surface plasticity to achieve bonding with the next layer [11,12,13,31,45,46]. If the material stiffens too rapidly, buildability may improve, but the interface may become weak because the fresh layer cannot properly merge with the previous one. If the mixture remains too fluid, interlayer contact may be improved, but deformation, loss of dimensional accuracy, and collapse may occur. Thus, the mechanical performance of printed concrete depends on a balance between shape retention and interfacial continuity. This balance is one of the main reasons why rheological behavior must be considered together with layer geometry when evaluating anisotropy.

**Figure 1:**
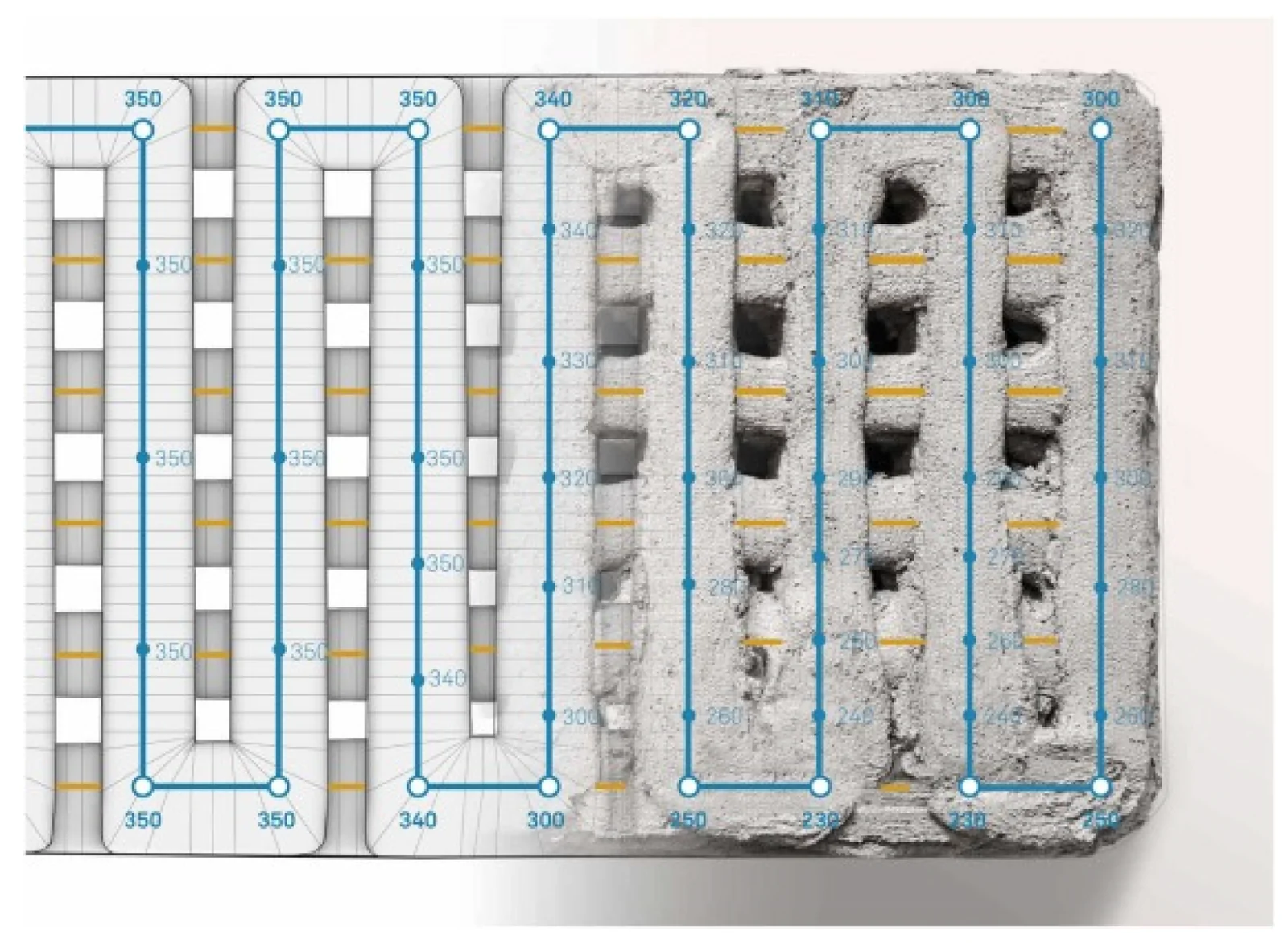
Bridge-scale 3D concrete printing with special geometry [47]. Licensed under CC BY 4.0.

The architectural arrangement of the printed path can either reduce or intensify anisotropic weakness. Toolpath design determines the orientation of filaments, the location of interfaces, the continuity of load-bearing paths, and the presence of voids or discontinuities between adjacent printed segments. Breseghello and Naboni [47] showed that toolpath-based design can be used to create carbon-efficient architectural structures by aligning material deposition with structural and geometric requirements. In such cases, geometry is not only a formal outcome of printing; it becomes a structural variable that affects stiffness, load transfer, and failure mode. Figure 1 illustrates a bridge-scale printed structure with a special geometry, where the printed arrangement contributes directly to the architectural and structural behavior of the element [47]. However, toolpath optimization cannot fully eliminate material anisotropy unless interlayer bonding, pore distribution, and filament continuity are also controlled.

Fiber incorporation adds another level to hierarchical anisotropy. Glass, steel, basalt, polypropylene, carbon, and other fibers can improve crack-bridging capacity, flexural resistance, and post-cracking behavior of cementitious materials, but extrusion tends to orient fibers along the printing direction [24,25,26,27,28,29,30,48,49,50,51]. This orientation may enhance the longitudinal response while leaving the transverse and interlayer directions less effectively reinforced. Therefore, the contribution of fibers depends on whether they bridge the weak interfaces or simply follow the filament axis. Recent studies on fiber-reinforced 3D printable concrete have shown that fiber content, aspect ratio, and fresh-state stability are strongly interrelated, while printed fiber geometry and reinforcement arrangement can alter fracture behavior and crack development [19,29,30,52,53]. Accordingly, fiber reinforcement should be designed with the hierarchical nature of 3DPC in mind: reinforcement must improve the weaker interfaces and off-axis response, not only the strength along the extrusion direction.

**Figure 2:**
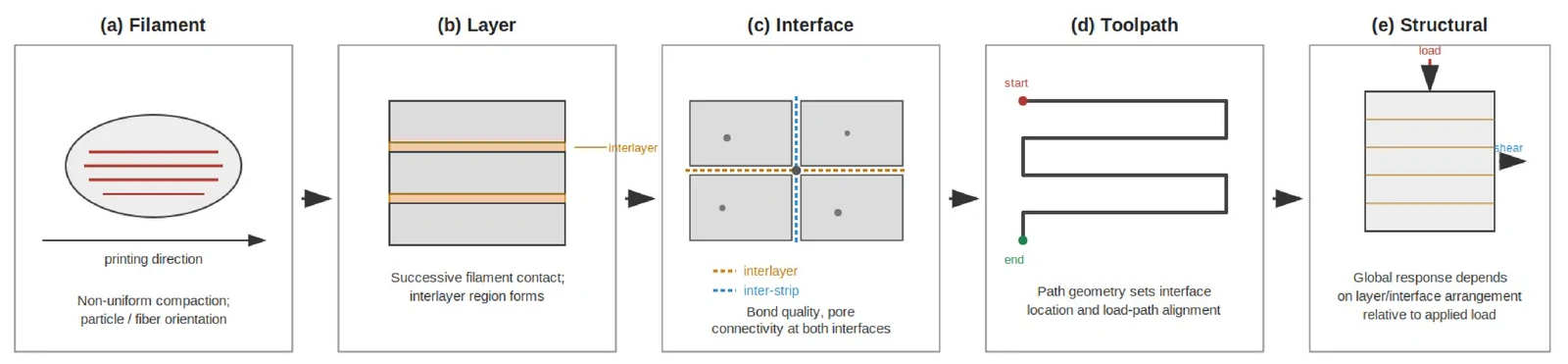
Hierarchical scales of anisotropy in 3D-printed concrete: (**a**) filament-scale particle/fiber orientation; (**b**) layer-scale formation of the interlayer region; (**c**) interface-scale interlayer and inter-strip bonding and pore connectivity; (**d**) toolpath-scale deposition geometry; (**e**) structural-scale load transfer across layers and interfaces. Original schematic.

Figure 2 summarizes this hierarchy schematically, showing how anisotropy accumulates from the filament scale to the structural scale through the layer, interface, and toolpath levels discussed above.

Overall, hierarchical anisotropy in 3DPC arises from the interaction between printing kinematics, fresh-state material response, interfacial bonding, and structural geometry. The filament core, filament surface, interlayer zone, inter-strip contact, and global toolpath each contribute differently to the final mechanical behavior. For this reason, reliable structural use of 3DPC requires more than optimizing mixture strength. It requires coordinated control of nozzle movement, layer geometry, rheology, interlayer time, reinforcement orientation, and toolpath design so that the printed architecture can transfer loads without creating preferential planes of weakness.

### 2.2. Pore Architecture

Porosity is one of the principal microstructural features governing the anisotropic behavior of 3D-printed concrete. Unlike conventionally cast concrete, which can be compacted by vibration or designed to consolidate within formwork, extrusion-based 3DPC is deposited without post-placement compaction. As a result, air voids may remain trapped within the filament body and, more critically, along the contact region between successive layers. These pores are not randomly distributed in the same manner as in cast concrete. Their shape, orientation, connectivity, and location are strongly affected by the extrusion process, nozzle geometry, layer height, deposition pressure, interlayer time, and the degree of contact between adjacent filaments [54,55,56].

The pore structure of 3DPC should therefore be considered as an architectural feature of the printed material rather than as a simple measure of total void content. Pores located inside the filament core mainly affect the intrinsic strength of the printed material, whereas pores concentrated at the interlayer region directly weaken the bond between layers and promote direction-dependent failure. When interfacial porosity increases, the effective contact area between successive layers decreases, stress transfer becomes less uniform, and cracks can propagate preferentially along the layer boundary. This explains why increases in interlayer porosity are frequently associated with reductions in compressive, tensile, and flexural performance [57,58,59].

Printing pressure and filament consolidation play an important role in controlling the formation of voids. Adequate pressure during extrusion can improve filament density and reduce internal defects, whereas insufficient pressure may produce poorly compacted filaments with irregular pore distribution [55,56]. However, excessive pressure or inappropriate nozzle height may disturb the shape of the deposited layer, promote lateral spreading, or alter the designed filament geometry. The offset distance between adjacent layers or filaments is also important. If the deposited path does not provide sufficient overlap or contact, voids may form between neighboring filaments, reducing interlayer bond strength and creating preferential paths for crack propagation and fluid ingress [59].

The time interval between consecutive layers further modifies local porosity and interfacial quality. As the surface of the previously deposited layer loses moisture and gains stiffness, the ability of the next layer to merge with it decreases. Longer interlayer intervals can therefore increase the probability of entrapped voids, weak contact zones, and discontinuities at the interface. Previous studies have shown that increasing the time gap between layers can markedly change void content and reduce bond quality, confirming that porosity in 3DPC is closely linked to the evolution of the fresh material during printing [60,61]. This effect is particularly relevant for anisotropy because the interlayer region becomes mechanically different from the filament core.

Void morphology is as important as void volume. Rounded and isolated pores generally produce lower stress concentration than elongated, flattened, or connected pores. In printed concrete, interfacial pores are often aligned or elongated along the layer boundary, which makes them more detrimental under tensile, flexural, and shear loading. Such pores reduce the compactness of the interlayer region and facilitate crack initiation at lower stress levels [62,63]. Therefore, the mechanical behavior of 3DPC cannot be interpreted only through total porosity. The location, shape, connectivity, and orientation of pores must also be considered, particularly when evaluating anisotropic strength and durability. Table 4 compiles porosity values reported separately for the filament core and the interlayer region, confirming that interlayer porosity can approach or exceed core porosity depending on the interlayer time gap.

The relationship between pore-scale architecture and macroscopic anisotropic performance operates through several coupled mechanisms across scales rather than a single, direct proportionality. At the micro-scale, interlayer pores act as local stress concentrators. A flattened or elongated void oriented parallel to the layer boundary produces stress amplification governed by pore aspect ratio and orientation relative to the applied load, rather than by porosity alone; this is consistent with the elongated, tri-axially oriented voids reported along the print direction and the more detrimental elongated interfacial pores discussed above [62,63,64]. This explains why two specimens with similar total interlayer porosity but differing pore shape can show markedly different tensile or flexural strength in the interlayer direction, while exhibiting comparable compressive strength, a direction in which blunt, rounded voids are comparatively less detrimental. At the meso-scale, the effective load-bearing cross-section of the interlayer plane is reduced in proportion to the interconnected pore area intersecting that plane. Because pore connectivity, rather than isolated pore volume, controls this reduction, interlayer regions with similar nominal porosity but higher connectivity—for example, resulting from prolonged interlayer time, as summarized in Table 4—present a smaller effective bonded area and correspondingly lower macroscopic strength [60,61,64]. At the macro-scale, this reduced and spatially non-uniform bonded area governs where cracks preferentially nucleate and how they propagate: cracks initiate at the most porous interlayer segments and follow the plane of least resistance rather than crossing the denser filament core, consistent with the tendency of 3DPC failure surfaces to follow interlayer boundaries under transverse loading. These three scales are coupled: printing parameters such as nozzle height, deposition pressure, and interlayer time set the pore-scale geometry [54,55,56], pore-scale geometry sets the meso-scale effective bonded area, and effective bonded area governs the macro-scale anisotropic strength ratios summarized in Table 3. A recent CT-informed statistical reconstruction and finite-element study demonstrated this coupling directly, showing that elongated macro-voids progressively cluster into a continuous weakened interlayer band as the interlayer time gap increases, and that this clustering governs a measurable size effect on predicted flexural strength [65]. Such explicit multiscale modeling frameworks, linking CT-derived pore geometry to macroscopic strength prediction, remain comparatively rare in the 3DPC literature and constitute a promising avenue for connecting microstructural observation to macroscopic design values.

Rheology is directly linked to pore architecture. A mixture with insufficient stability may deform after deposition and produce irregular layer geometry, whereas an overly stiff mixture may prevent proper contact between consecutive layers. Pumpability, extrudability, yield stress development, and thixotropic build-up must therefore be balanced to minimize internal voids while preserving interlayer bonding [11,12,13,31,42]. It records how the mixture flowed through the nozzle, how it was deposited, how it contacted the previous layer, and how its surface evolved before the next layer was placed.

### 2.3. Anatomy of a Cold Joint in 3D Printing

A cold joint in 3DPC forms when the interface between two consecutively deposited layers fails to develop sufficient physical and chemical continuity. Although the term is commonly used in conventional concrete for delayed casting, its meaning in 3D printing is more specific. In printed concrete, a cold joint is not only a temporal discontinuity; it is a weak interfacial zone produced by the combined effects of surface drying, structural build-up, reduced plasticity, incomplete contact, entrapped air, and interrupted hydration across the layer boundary [33,61,66].

The formation of a cold joint begins immediately after a filament is deposited. The exposed surface starts losing moisture, the cementitious matrix undergoes early flocculation and structural build-up, and the surface becomes progressively less deformable. If the next layer is placed while the first layer still has sufficient surface plasticity, the two layers may merge and develop a relatively continuous interface. If the deposition is delayed, the new layer can rest on a surface that is already stiff, dry, or partially set. Under this condition, the interface becomes a plane of reduced contact, higher porosity, and weaker mechanical interlocking. The resulting discontinuity can govern the failure mode of the printed element.

Cold joints are especially harmful because they change the stress distribution within the printed structure. Instead of transferring stresses continuously through the filament body and across the bonded interface, the load is redirected around weak contact regions and interfacial voids. This produces local stress concentration and promotes crack initiation along the layer boundary. Under shear and flexural loading, the cold joint may act as a preferential crack path, leading to interlayer debonding or sliding. In contrast, specimens with better interlayer continuity are more likely to develop cracks through the material body, which usually indicates a stronger and more integrated structural response [44].

The severity of a cold joint depends on the time interval between layers, the rate of surface moisture loss, mixture setting behavior, environmental exposure, and the rheological evolution of the material. Fast-setting mixtures may improve early buildability, but they can also accelerate the loss of interfacial plasticity and increase the risk of weak bonding if the printing process is interrupted. Similarly, long waiting times between layers can allow more surface drying and create a rough but poorly bonded interface. In such cases, the interface may contain numerous voids and discontinuities, reducing the integrity of the printed element [33,61,66]. Table 5 compiles interlayer bond strengths reported as a function of interlayer time gap across several studies, highlighting the pronounced strength loss associated with longer waiting times between layers.

Cold joints are closely related to anisotropy because they introduce preferential planes of weakness into the printed body. Their influence is not limited to mechanical strength. They may also increase permeability, facilitate moisture and ion transport, and accelerate durability-related degradation when the printed element is exposed to chloride ingress, carbonation, sulfate attack, or freeze–thaw cycles. Therefore, cold joints should be evaluated not only as local defects but also as structural and durability-critical features of 3DPC.

**Figure 3:**
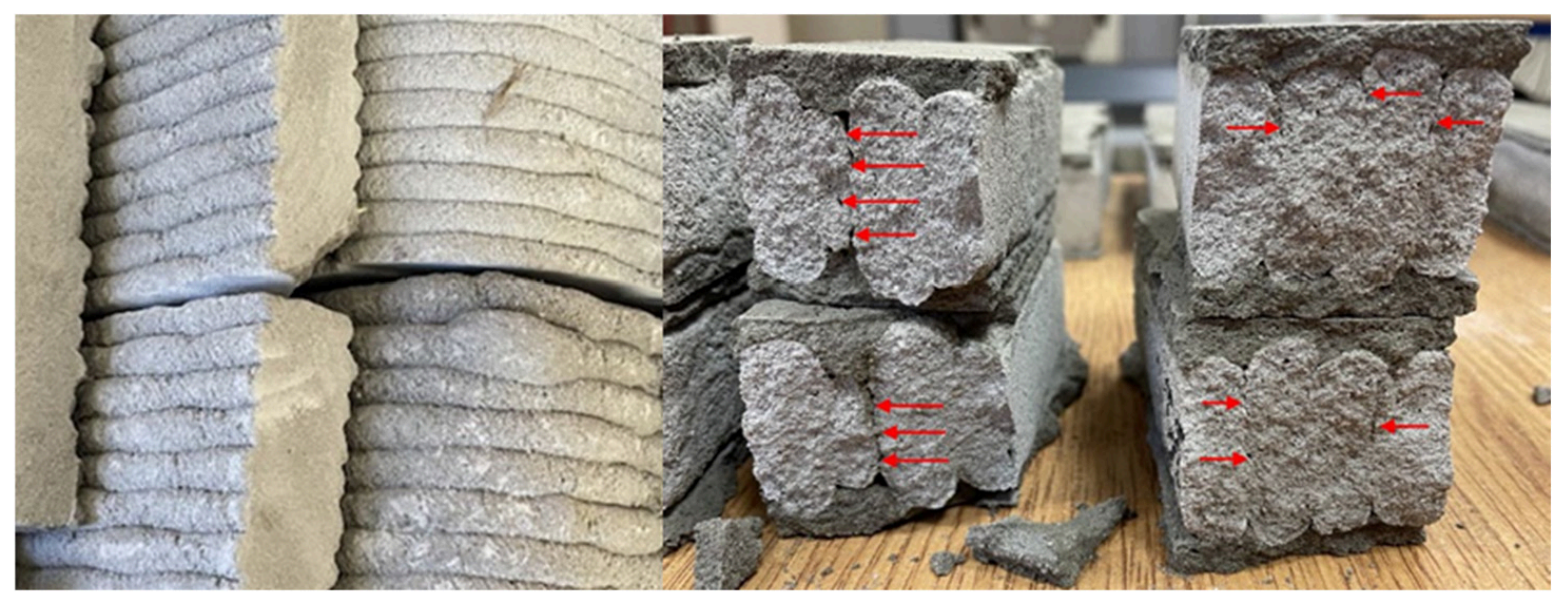
Macro-scale example of a cold joint in 3DPC; red arrows indicate discontinuities at the interlayer interface [44]. Licensed under CC BY 4.0.

Figure 3 shows a macro-scale example of a cold joint, where the red arrows indicate visible discontinuities at the interlayer interface [44]. Such discontinuities illustrate how interrupted deposition and insufficient interlayer bonding can transform the printed interface into a weak plane. From a structural perspective, this weak plane may control crack development, reduce load-transfer capacity, and intensify the anisotropic response of the printed concrete element.

### 2.4. Preferential Orientation of Constituents

The preferential orientation of constituents is one of the main material-level causes of anisotropy in extrusion-based 3DPC. During extrusion, cement particles, fine aggregates, pores, and fibers are subjected to shear and directional flow through the nozzle. This process can rearrange the internal structure of the fresh mixture before deposition. As a result, the printed filament does not have the same internal fabric in all directions. The material may become denser and better aligned along the extrusion direction, while the transverse and interlayer directions remain more vulnerable to cracking and debonding.

Fiber addition is commonly used to improve the mechanical performance of printable cementitious materials, particularly tensile resistance, flexural strength, crack-bridging capacity, and post-cracking behavior [69,70,71]. However, in 3DPC the role of fibers is more complex than in cast concrete. In conventionally cast mixtures, fibers may be distributed more randomly if the mixture is properly mixed and placed. In extrusion-based printing, the flow field inside the hose and nozzle tends to orient fibers along the direction of material movement. This alignment can improve the longitudinal response of the printed filament, but it may not provide the same reinforcement efficiency across the interlayer region. Therefore, fiber-reinforced 3DPC may still exhibit direction-dependent mechanical performance even when the overall strength is improved.

The effect of fiber orientation depends on fiber type, aspect ratio, volume fraction, stiffness, surface condition, and compatibility with the fresh matrix. Ma et al. reported that the incorporation of basalt fibers at an optimized dosage improved the mechanical performance of printed cementitious composites [27]. This improvement can be attributed to crack bridging, restriction of crack opening, and enhanced energy dissipation during fracture. Nevertheless, the improvement is not necessarily uniform in all loading directions. If the fibers are mainly aligned with the extrusion path, their ability to bridge cracks crossing the interlayer boundary may be limited. In such cases, the printed element may show higher resistance along the filament direction but remain weaker under transverse tension, flexure, or interlayer shear.

Recent studies on fiber-reinforced 3D printable concrete also show that fiber content and aspect ratio influence not only hardened strength but also fresh-state stability, extrudability, and buildability [72]. Excessive fiber content may disturb flow through the nozzle, reduce filament continuity, increase the risk of blockage, or create local defects. Conversely, insufficient fiber content may not provide enough crack-bridging capacity to compensate for weak interlayer bonding. The design of fiber-reinforced 3DPC must therefore consider both printing performance and structural response. Fiber reinforcement should be evaluated according to whether it improves the weaker directions of the printed element, not only whether it increases compressive or flexural strength under a single loading configuration.

The geometry and spatial arrangement of reinforcement are also important. Studies on printed fiber geometry and content have shown that fracture behavior is sensitive to how reinforcement is distributed within the cementitious body [19]. These findings are relevant to 3DPC because the printed process itself imposes directionality on both the matrix and the reinforcement. For this reason, the use of fibers or discrete reinforcement should be considered as part of the printed material architecture rather than as an independent strengthening method.

Liu et al. showed that extrusion-based printed concrete could display a clear anisotropic response when compared with cast concrete, with compressive performance varying according to the loading direction [43]. This observation supports the view that anisotropy in 3DPC is not caused only by weak interlayer bonding. It also arises from the internal orientation of constituents generated during extrusion. Therefore, preferential orientation must be considered together with pore architecture, cold joints, and interlayer bonding when interpreting the mechanical behavior of printed concrete.

## 3. Chemical Reasons for Anisotropic Weakness

### 3.1. Disrupted Hydration

Hydration plays a central role in the development of strength and durability in cement-based materials. In 3DPC, however, hydration does not occur under the same boundary conditions as in conventionally cast concrete. The printed filament is exposed to air immediately after deposition, and its surface may lose moisture before the next layer is placed. This condition is especially important at the interlayer region, where bonding depends on the ability of hydration products to develop continuously across the contact zone.

During normal hydration, calcium silicate hydrate and calcium hydroxide progressively form and fill part of the capillary pore space. This process contributes to strength gain, stiffness development, and refinement of the pore structure. If early moisture loss occurs before sufficient hydration products have formed, the interfacial region may remain more porous and less cohesive [73,74]. In 3DPC, this risk is higher because each printed layer has an exposed surface, and the time interval between consecutive layers controls the degree of surface drying, stiffness development, and contact quality. As a result, the interlayer zone may develop a microstructure that differs from the filament core.

The disruption of hydration at the interface can weaken both mechanical and durability performance. A poorly hydrated interlayer region may contain fewer continuous C-S-H bridges, higher capillary porosity, and more disconnected contact points between layers. These features reduce bond strength and facilitate crack propagation along the interface. They may also increase the transport of water, oxygen, chloride ions, sulfate ions, and carbon dioxide through the printed element. Therefore, hydration disruption should not be considered only as a chemical issue; it is directly connected to anisotropic mechanical behavior and durability loss.

The chemistry of the pore solution and the presence of aggressive ions can further alter the interfacial microstructure. Sulfate ions may penetrate through connected pores and react with hydration products to form expansive phases such as ettringite and gypsum [75]. These reactions can increase internal stress, disturb the cement matrix, and accelerate cracking when sufficient moisture and transport pathways are present. In printed concrete, connected interlayer pores and weak contact zones may provide easier access for such ions, making the chemical degradation process direction-dependent.

The rheological state of the mixture before and during extrusion also affects hydration development. The use of supplementary cementitious materials, chemical admixtures, viscosity-modifying agents, and water-reducing admixtures can change flocculation, setting behavior, early structural build-up, and the distribution of hydration products [76]. These changes influence both printability and the quality of interlayer bonding. SEM- and EDS-based studies on cementitious materials confirm that the morphology and distribution of C-S-H are closely associated with pore refinement and mechanical performance [32]. This is particularly relevant for 3DPC because the interlayer region may not receive the same moisture, compaction, and hydration conditions as the filament core.

### 3.2. Direction-Dependent Durability

The durability of 3DPC is strongly affected by its anisotropic material architecture. In printed elements, transport properties are not governed only by total porosity; they also depend on the orientation and connectivity of pores, the continuity of the interlayer region, and the direction of exposure relative to the printed layers. Therefore, samples extracted parallel and perpendicular to the printing direction may show different durability responses, especially under freeze–thaw exposure, water absorption, chloride ingress, and carbonation [77].

The extrusion process can increase porosity, reduce interlayer bond quality, and create preferential pathways for moisture and ion transport [78,79,80]. These features are closely linked to durability degradation. When pores and weak interfaces are aligned with the printed layers, aggressive agents may move more easily along the interlayer region than through the denser filament core. This can produce direction-dependent deterioration that is not normally observed in the same way in well-compacted cast concrete.

Freeze–thaw resistance is particularly sensitive to pore structure and moisture transport. In 3DPC, the absence of vibration, the presence of interlayer voids, and the orientation of printed layers can influence the degree of saturation and the development of internal hydraulic pressure during freezing. Horizontally and vertically cored specimens may therefore exhibit different resistance to freeze–thaw damage because the direction of water movement and the orientation of defects are different [77]. This explains why durability assessment of 3DPC should consider printing direction and layer orientation, not only mixture composition.

Drying shrinkage is another durability-related issue. Unlike cast concrete, which is protected by formwork during the early stage, printed concrete has exposed surfaces immediately after deposition. This exposure can accelerate moisture evaporation, increase shrinkage gradients, and contribute to micro-cracking near the surface and interlayer region [81]. When shrinkage-induced micro-cracks intersect with existing pores or weak interfaces, the transport of aggressive agents may become easier. Consequently, shrinkage, porosity, interlayer bonding, and durability should be interpreted as connected phenomena rather than isolated properties.

The printed pore network also affects chloride ingress. Compared with conventional concrete, 3DPC often contains higher and more connected porosity, especially near layer boundaries [68]. If chloride ions penetrate through these connected pathways, they may remain within the pore system and increase the risk of reinforcement corrosion when metallic reinforcement is present. Even in unreinforced printed elements, chloride ingress remains important because it reflects the permeability and durability quality of the printed matrix. Thus, the durability of 3DPC depends not only on the chemistry of the binder but also on the printed architecture of the pore system.

### 3.3. Surface Carbonation and Sulfate Attack

Carbonation and sulfate attack are important degradation mechanisms for cement-based materials, and their effects can be intensified by the anisotropic pore structure of 3DPC. In conventional concrete, carbonation is controlled by the penetration of carbon dioxide through the pore network and its reaction with calcium-bearing hydration products. In 3DPC, the interlayer region may provide preferential pathways for carbon dioxide because of higher porosity, incomplete contact, and weaker hydration continuity between layers. As a result, carbonation depth may vary depending on the direction of exposure and the orientation of the printed layers.

The carbonation of 3DPC is closely linked to interlayer time, surface moisture condition, layer height, and nozzle speed [67]. A longer interval between layers can increase surface drying and reduce interfacial continuity, which may accelerate carbon dioxide penetration along the layer boundary. Previous studies have reported that carbonation depth in printed concrete can increase with longer printing intervals, and that printed specimens may exhibit greater carbonation depth than cast specimens under comparable exposure conditions [82,83]. These observations indicate that carbonation in 3DPC is not controlled only by binder chemistry. It is also governed by the physical continuity of the printed interface.

Transport pathways and pore connectivity similarly influence sulfate attack. Sulfate ions can enter the cementitious matrix through connected pores and react with hydration products to form expansive products such as ettringite and gypsum [75,84]. These reactions may cause expansion, cracking, softening, and loss of strength. In printed concrete, connected interlayer pores and cold-joint regions can increase the accessibility of sulfate ions to the interior of the element. Therefore, sulfate attack may become more severe when the printed interface is poorly bonded or contains continuous voids.

Initial curing is critical for improving resistance to chemical attack. Adequate curing allows hydration products to develop, refines the pore structure, and reduces transport pathways before exposure to aggressive environments. Studies on cement-based materials exposed to sulfate environments have shown that a sufficient initial curing period is necessary to improve resistance to degradation [85]. For 3DPC, this point is particularly important because the printed layers are exposed from the moment of deposition and may dry rapidly if curing is delayed or insufficient. Proper curing must therefore be treated as part of interfacial quality control, not only as a general durability measure.

Overall, carbonation and sulfate attack in 3DPC should be interpreted through the combined effects of binder chemistry, pore connectivity, interlayer continuity, and exposure direction. The anisotropic structure of printed concrete can create preferential ingress paths, while disrupted hydration and weak interlayer bonding can reduce the resistance of the interface to chemical degradation. This interaction explains why durability performance may differ significantly between printed and cast concrete, even when similar binder systems are used.

### 3.4. Porosity-Controlled Transport and Durability

Porosity was discussed earlier as a structural feature of printed architecture; however, its role in chemical durability requires separate attention. In 3DPC, porosity controls the movement of water, gases, and dissolved ions through the printed element. The durability problem is therefore not only the presence of pores, but also their connectivity, orientation, and position relative to the interlayer region.

Low porosity in conventional concrete is often achieved through proper compaction, vibration, or self-consolidation. In contrast, 3DPC is deposited without vibration, and the final pore structure is governed by extrusion pressure, nozzle geometry, layer height, mixture rheology, and interlayer contact [64,68]. Poorly compacted filaments may contain internal voids, while weak interfaces may contain flattened or elongated pores along the layer boundary. These interfacial pores can provide preferential paths for water absorption and ion ingress.

The effect of porosity on freeze–thaw durability is particularly important. Connected pores can increase water uptake and raise the degree of saturation. When freezing occurs, the expansion of water and the movement of unfrozen water through the pore system can generate internal pressure. If the pore network is poorly distributed or if interlayer defects concentrate moisture, damage may develop preferentially along the layer boundary. This mechanism links pore architecture directly to the anisotropic freeze–thaw response of 3DPC.

Porosity also influences chloride transport. Printed elements with higher interlayer porosity may allow chloride ions to penetrate more rapidly than dense cast specimens [68]. The problem is more severe when the pore network is continuous along the printed layer direction. In such cases, the interlayer region does not merely act as a weak mechanical plane; it also becomes a transport channel. This dual role explains why interlayer porosity affects both mechanical anisotropy and long-term durability.

Interlayer pauses can further increase porosity and degrade interfacial microstructure. When the time gap between layers increases, the previously deposited surface becomes less plastic and less capable of merging with the next layer. This condition can produce voids, weak contact zones, and reduced flexural performance [86]. Similarly, nozzle size and extrusion conditions may change the density and pore structure of printed foam concrete elements, affecting both weight and mechanical behavior [87]. These observations confirm that porosity in 3DPC is strongly process-dependent.

Water absorption is also affected by the external surface texture of printed concrete. The visible layer contours increase the exposed surface area and may promote water retention along the printed grooves. Moreover, pore morphology may differ between the outer surface, filament core, and interlayer region. When water exposure occurs from the side of the printed element, flattened or aligned pores near the layer boundary can facilitate directional transport [60,88,89]. Therefore, durability evaluation of 3DPC should account for both the internal pore structure and the printed surface morphology.

A critical evaluation of the chemical and durability studies cited above reveals a significant methodological heterogeneity that limits direct comparison across the literature. Reported carbonation depths, sulfate resistance data, and freeze–thaw performance were obtained using printers, nozzle geometries, mixture proportions, and specimen extraction protocols that differ substantially between research groups [64,67,68,73,74,75,76,77,78,79,80,81,82,83,84,85,86,87,88,89]. Interlayer time, in particular, is rarely standardized: values ranging from a few seconds to several hours are used to represent “delayed” deposition, which makes it difficult to establish threshold values applicable across different material systems. Moreover, most durability assessments rely on small-scale coupon or cored specimens tested under accelerated laboratory exposure, and it remains uncertain whether these results can be extrapolated to full-scale printed elements exposed to real environmental cycles over years rather than weeks. Future studies should adopt more standardized interlayer-time protocols and report printer-specific parameters in sufficient detail to allow meta-analysis across independent investigations.

## 4. Physical Reasons for Anisotropic Weakness

### 4.1. Lack of Mechanical Interlocking

Mechanical interlocking between printed layers is one of the main physical mechanisms controlling the anisotropic response of 3DPC. In conventionally cast concrete, fresh material is usually placed as a continuous volume and may be compacted by vibration or self-consolidation. In extrusion-based printing, however, each layer is deposited on a previously printed surface that has already started to lose moisture, build internal structure, and resist deformation. As a result, bonding between layers depends not only on chemical adhesion but also on the ability of the fresh layer to physically penetrate, deform, and interlock with the surface irregularities of the preceding layer.

The interlayer bond is strongly governed by the fresh-state rheology of the printable mixture. Yield stress, plastic viscosity, thixotropic build-up, open time, and structural recovery after extrusion determine whether the deposited filament can maintain its shape while still forming an intimate contact with the next layer [45,46,90,91]. A mixture with rapid thixotropic build-up may provide good buildability, but it can also reduce interlayer bonding if the surface becomes too stiff before the subsequent layer is placed. Conversely, a mixture with insufficient structural build-up may improve local contact between layers but may suffer from excessive deformation, loss of dimensional accuracy, or collapse under self-weight. Therefore, the mechanical performance of 3DPC depends on a balance between shape stability and interfacial deformability.

The lack of mechanical interlocking becomes particularly critical when the interlayer surface is smooth, dry, or poorly compressed by the next deposited filament. Under such conditions, the interface behaves as a preferential weak plane. The load is then transferred through a reduced contact area, and stress concentrations develop around pores, surface discontinuities, and unbonded regions. This mechanism explains why interlayer debonding, sliding, and crack propagation along the layer boundary are frequently observed under flexural, tensile, and shear loading [59,92]. In compression, the effect may be less obvious at low stress levels, but failure can still be governed by the combined response of the filament core, interlayer region, and inter-strip contacts when the printed element is loaded unfavorably relative to the deposition direction.

Nozzle geometry, layer height, deposition pressure, and surface texture also influence mechanical interlocking. A properly adjusted nozzle height may slightly compress the fresh filament against the previous layer and increase the real contact area. However, excessive compression can disturb the layer geometry or squeeze material laterally, while insufficient compression leaves voids and weak contact zones. Similarly, roughened or geometrically modified interfaces can improve physical interlock, but only if the fresh material remains deformable enough to fill surface asperities. This is why interlocking cannot be treated as a purely geometric issue; it is governed by the combined action of rheology, printing time, surface condition, and deposition mechanics.

The anisotropic weakness caused by insufficient interlocking is therefore a direct consequence of the printing sequence. Each new layer is required to behave in two conflicting ways: it must be stiff enough to support the following layers, but plastic enough to bond with the previous one. If this balance is not achieved, the printed structure develops laminated behavior, where the layer boundary becomes mechanically weaker than the filament body. Improving mechanical interlocking requires coordinated control of mixture rheology, interlayer time, nozzle height, deposition pressure, and surface morphology rather than relying on mixture strength alone.

It should be noted that the studies underpinning these conclusions employ markedly different testing configurations to quantify interlayer bond strength, including direct tensile, splitting tensile, flexural, and shear-slant setups, each of which imposes a different stress state on the interface and is therefore not directly comparable [45,46,59,90,91,92]. Specimen geometry, loading rate, and the assumed failure plane also vary between studies, which may partly explain the wide scatter in reported interlayer-to-bulk strength ratios in the literature. Without a common testing protocol, it is difficult to isolate the individual contribution of rheological parameters (yield stress, thixotropic build-up) from that of process parameters (nozzle height, deposition pressure) to interlayer bond development, and this limitation should be considered when comparing quantitative interlocking results across the reviewed studies.

### 4.2. Lack of Reinforcement in 3DPC

The integration of reinforcement remains one of the most difficult physical challenges in 3DPC. Conventional reinforced concrete relies on continuous steel bars, stirrups, meshes, or cages to provide tensile capacity, ductility, crack control, and structural redundancy. In extrusion-based 3DPC, the layer-by-layer process interrupts the straightforward placement of such reinforcement. The material is deposited continuously along a toolpath, while conventional reinforcement usually requires pre-positioning, accurate cover control, mechanical anchorage, and compatibility with the final load path. This mismatch between printing kinematics and reinforcement placement limits the direct transfer of conventional reinforced concrete practice to printed structures.

Several studies have demonstrated the feasibility of printable cementitious or geopolymer mixtures without conventional reinforcement, and some unreinforced printed structural forms have been successfully designed using geometry-dominated load transfer [14,15]. Nevertheless, the absence of continuous reinforcement remains a major limitation for structural elements subjected to bending, tension, shear, impact, thermal gradients, or long-term service loading. In such cases, anisotropy becomes more critical because the weak interlayer regions are not crossed by reinforcement capable of transferring tensile stresses or controlling crack opening. Therefore, unreinforced 3DPC may perform adequately in compression-dominated geometries but remains vulnerable when tensile or flexural stresses develop across layer boundaries.

At large scale, reinforcement strategies for 3DPC can generally be grouped into pre-installed reinforcement, post-installed reinforcement, embedded reinforcement during printing, textile or mesh-based reinforcement, fiber reinforcement, and hybrid systems [93,94,95]. Each method has advantages and limitations. Pre-installed reinforcement can provide continuity and ductility, but it may obstruct nozzle movement and restrict geometric freedom. Post-installed reinforcement avoids interference during printing, but it may require drilling, grouting, anchorage, or additional labor. Embedded reinforcement during printing is attractive in principle, yet it requires precise synchronization between material deposition and reinforcement placement. Textile, FRP, or mesh systems can improve crack control and interlayer stress transfer, but their effectiveness depends on placement accuracy, bond quality, and compatibility with the printed matrix.

The lack of reinforcement is closely connected to anisotropic weakness because printed layers create directional planes of reduced tensile and shear resistance. Without continuous reinforcement crossing these planes, cracks can initiate and propagate along the interlayer region with limited resistance. This problem is particularly important for elements in which the deposition direction does not coincide with the principal tensile stress direction. In such cases, the printed geometry may guide the load path, but it cannot fully compensate for insufficient tensile capacity at the interface. Reinforcement must therefore be designed not only to increase load-bearing capacity but also to bridge the weak directions introduced by the printing process.

**Figure 4:**
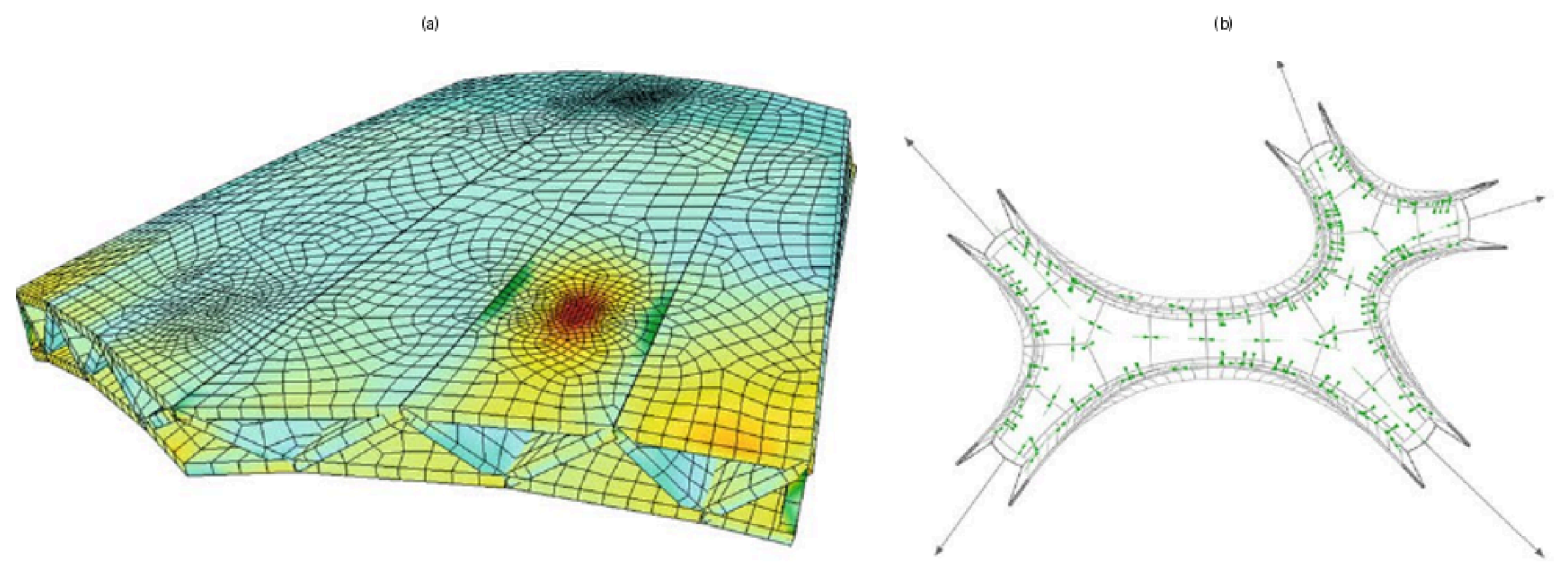
3DPC bridge: (**a**) finite element stress analysis and (**b**) internal truss-like force-vector diagram, adapted from Bhooshan et al. [96] (Figures 23a and 24c therein), licensed under CC BY 4.0.

Geometry-based design has been used to partially compensate for the absence of conventional reinforcement. Bhooshan et al. [96] examined a 3D-printed concrete bridge using finite element analysis and internal truss-like patterns to support the structural concept (Figure 4). Such approaches are valuable because they exploit the freedom of additive manufacturing to place material where it is structurally needed. However, geometry alone cannot eliminate the material-level weakness associated with interlayer bonding, lack of ductility, and crack control. A printed truss pattern may improve stiffness and distribute stresses more efficiently, but the interfaces within the printed material remain potential weak zones unless they are properly bonded or reinforced.

**Figure 5:**
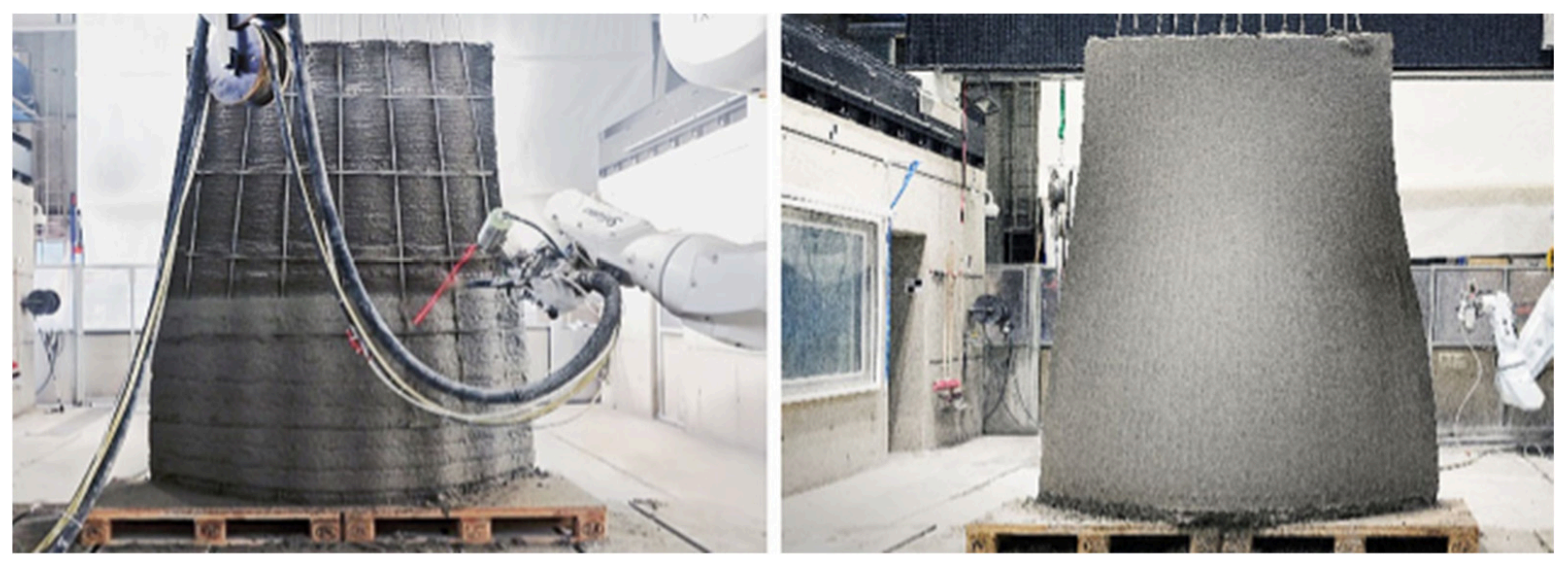
Reinforced wall produced using a shotcrete-based 3DPC method, adapted from [95]. Licensed under CC BY 4.0.

Shotcrete-based or hybrid printing approaches provide another route for incorporating reinforcement. In one example, a sprayed 3DPC process with an inclined nozzle was used over a reinforced wall system, showing that reinforcement and digital deposition can be combined in specific construction scenarios (Figure 5) [95,97]. This type of method may be useful for strengthening, repair, or wall construction where reinforcement is already present. However, it also introduces practical limitations, including additional labor, equipment complexity, quality control requirements, surface rebound, material loss, and dependence on operator or robotic precision. Therefore, such solutions should be evaluated not only by their mechanical benefit but also by their constructability and repeatability.

**Figure 6:**
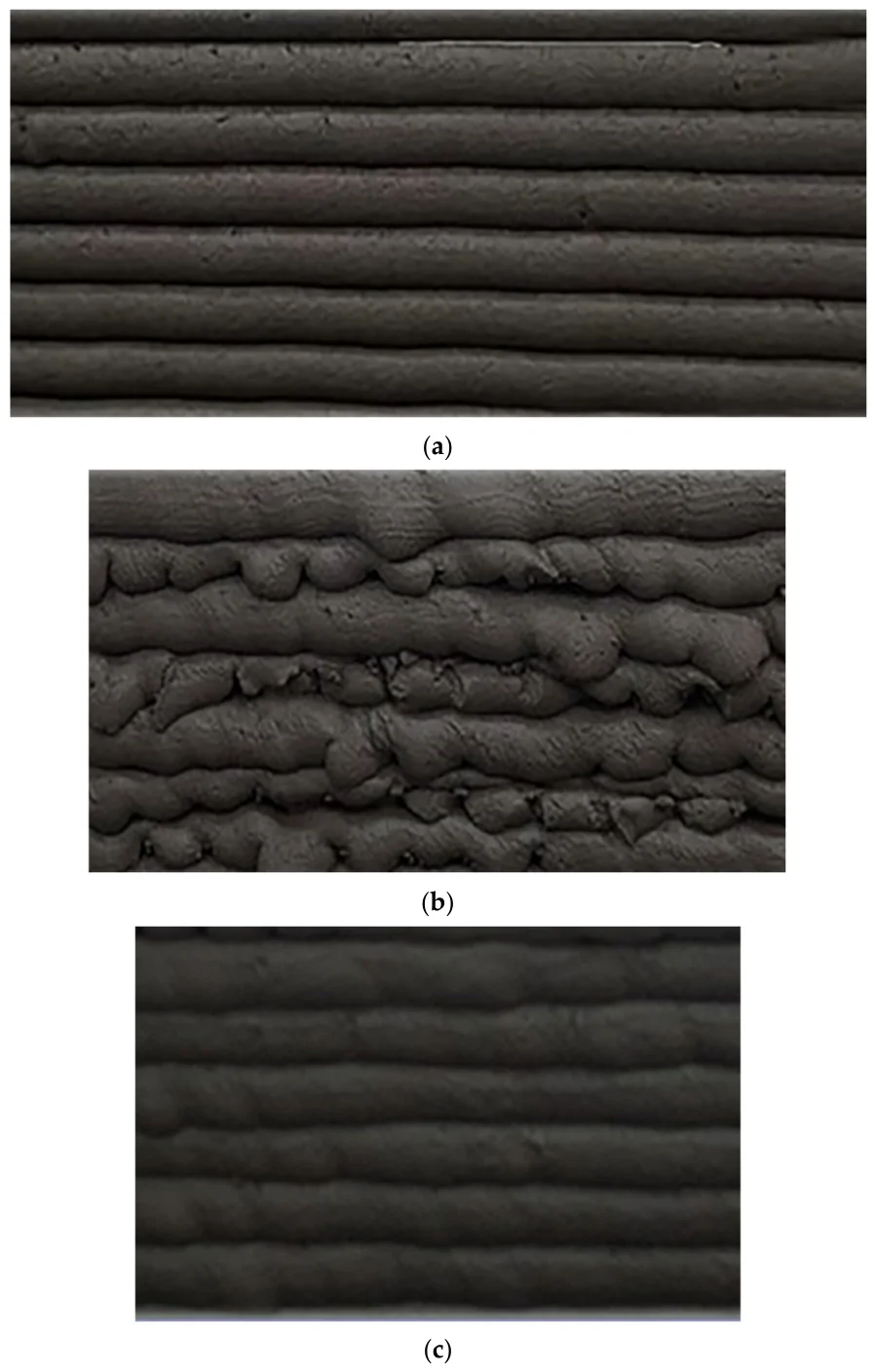
FRP-reinforced 3DPC under different extrusion conditions: (**a**) concrete extrusion at 40 mm/s without FRP, (**b**) extrusion at 30 mm/s with FRP, and (**c**) extrusion at 40 mm/s with FRP [97]. Licensed under CC BY 4.0.

FRP and textile reinforcement have attracted increasing attention because they can provide corrosion resistance, high tensile capacity, and compatibility with thin or geometrically complex printed elements. Sun et al. [97] investigated FRP-reinforced 3DPC and showed that printing parameters, including extrusion speed, affect the quality of the reinforced printed element (Figure 6). When properly integrated, FRP reinforcement can contribute to tensile resistance and reduce the severity of anisotropic weakness by bridging or restraining crack development across vulnerable regions. However, its performance depends on the bond between FRP and the cementitious matrix, the position of the reinforcement relative to the interlayer plane, and the ability of the printing process to maintain continuous contact without creating voids around the reinforcement.

Reinforcement effectiveness in 3DPC is ultimately governed by whether a given strategy bridges the weak interlayer plane or simply adds material to it—a distinction borne out by several recent studies. FRP has proven effective on both fronts: in-process dual-nozzle embedment of FRP grids substantially improves flexural strength, energy absorption, and ductility, and pull-out and bond tests on FRP bars confirm that reinforcement can meaningfully improve load transfer even though bond strength in 3DPC remains lower than in mould-cast concrete, primarily due to printing direction and fiber volume fraction rather than any deficiency in the FRP itself [98,99]. Yet the same mechanisms that make reinforcement effective can also undermine it: embedding a grid during printing introduces voids that weaken adjacent-layer bonding, and interlayer bond strength in multi-material printed systems (alkali-activated concrete, normal concrete, and engineered cementitious composites) has been shown to decline by up to 32.25% as the gap between depositions grows, with functionally graded specimens more sensitive to delay than homogeneous ones [98,100]. This tension is resolved, rather than merely illustrated, in functionally graded printed plates, where positioning an ECC layer specifically to bridge the weaker regions of the cross-section improved load-bearing and deformation capacity beyond what added material volume alone would predict [101]. Reinforcement in 3DPC therefore behaves less as a bulk property than as a targeted, position-dependent intervention against interlayer weakness.

**Figure 7:**
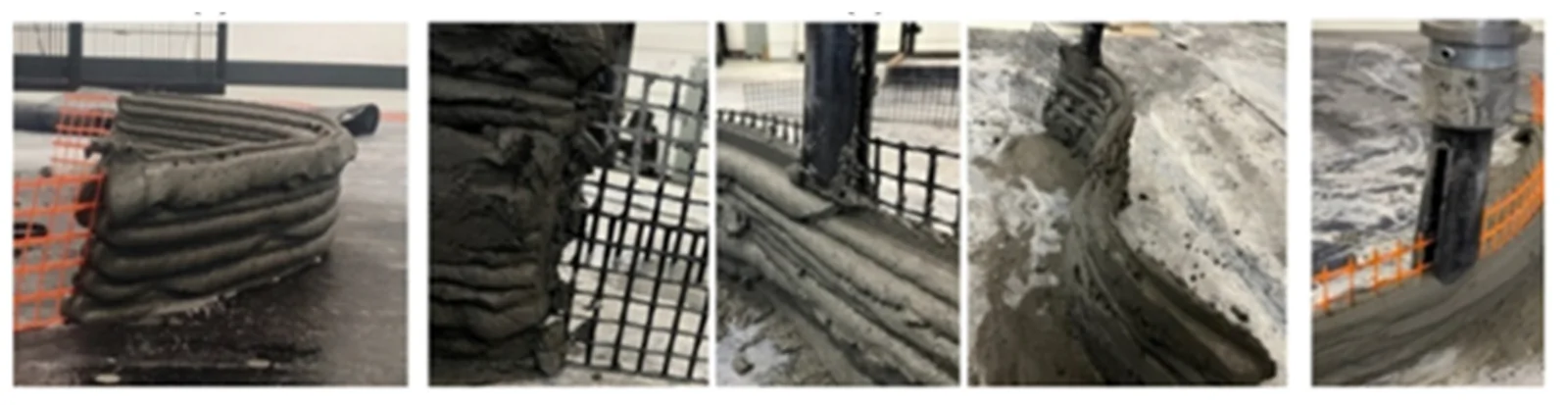
Steel textile reinforcement method for 3DPC [102]. Licensed under CC BY 4.0.

Textile reinforcement is another promising strategy. Ramesh et al. [102] used steel textile reinforcement in 3DPC and showed that textile systems can enhance the mechanical contribution of the printed element by improving crack distribution and stress transfer (Figure 7). Textile reinforcement may be particularly useful because it can be aligned with regions where tensile stresses are expected. Nevertheless, if the textile remains parallel to the printing direction and does not effectively bridge the weaker interlayer zones, anisotropy may persist. The reinforcement layout must therefore be coordinated with the expected stress field and the printed layer arrangement.

Discrete fibers, chopped fibers, and minibar-type reinforcement can also contribute to crack control and energy dissipation. Their advantage is that they can be incorporated into the printable mixture without requiring a separate placement operation. However, as discussed earlier, extrusion tends to orient fibers along the material flow direction. This may improve longitudinal performance but may not fully compensate for weakness across the layer interface. Recent studies on fiber-reinforced and printed cementitious systems indicate that fiber content, aspect ratio, geometry, and distribution influence both fresh-state printability and hardened fracture behavior [16,29,30,31]. Thus, discrete reinforcement should be considered as a complementary approach rather than a complete substitute for continuous reinforcement in structural 3DPC.

Overall, the lack of reinforcement in 3DPC is not simply a construction detail; it is a structural limitation that amplifies anisotropic weakness. The printed element contains direction-dependent interfaces, while the absence of continuous reinforcement reduces its capacity to redistribute tensile stresses and control crack propagation. Reliable structural application of 3DPC therefore requires reinforcement strategies that are compatible with printing kinematics, preserve geometric freedom, maintain interlayer bonding, and provide tensile continuity across the weaker directions of the printed material.

### 4.3. Fiber Reinforcement

Fiber reinforcement is one of the most widely investigated approaches for improving the mechanical performance of 3D-printed concrete, particularly in tension, flexure, crack control, and post-cracking resistance [71]. In printed cementitious materials, however, the contribution of fibers is governed not only by fiber type and dosage but also by their orientation, dispersion, aspect ratio, interaction with the nozzle, and bond with the surrounding matrix. This makes fiber reinforcement more complex in 3DPC than in conventionally cast fiber-reinforced concrete.

During extrusion, the material experiences shear flow inside the hose and nozzle. This flow tends to align short fibers along the printing direction, especially when the fibers have a high aspect ratio or when the nozzle geometry promotes directional movement [103,104]. Such alignment may improve the mechanical response parallel to the filament axis, but it may not provide the same benefit in the transverse or interlayer directions. Therefore, fiber addition can increase the overall tensile or flexural capacity while the printed element still remains anisotropic. This distinction is important because the weakest direction in 3DPC is often associated with the interlayer region rather than the filament core.

The effectiveness of fibers depends on whether they can bridge cracks across the weak regions of the printed element. If fibers are mainly aligned within the filament body, they may delay crack opening along the extrusion direction but provide limited resistance to interlayer debonding. Conversely, if the fiber arrangement allows crack bridging across the interlayer zone, the reinforcement can improve stress transfer and reduce the severity of directional weakness. For this reason, fiber-reinforced 3DPC should be evaluated not only by compressive or flexural strength values, but also by fracture path, crack width development, residual strength, and failure mode.

Short fibers have been proposed as partial alternatives to conventional reinforcement in 3DPC because they can be mixed directly into the printable matrix and deposited together with the cementitious material [105]. This is practically attractive because it does not require a separate reinforcement placement stage. Nevertheless, short fibers cannot fully replace continuous reinforcement in structural elements where tensile continuity, anchorage, ductility, and crack control are required over large spans or critical sections. Their role is more realistic as a complementary reinforcement strategy that improves crack resistance and energy dissipation at the material scale.

Polypropylene fibers are frequently used to reduce plastic shrinkage cracking and improve early-age crack control in cementitious systems [106]. In 3DPC, this function is particularly relevant because printed layers are exposed immediately after deposition, and the absence of formwork can accelerate surface moisture loss. However, polypropylene fibers generally have a lower elastic modulus than steel, basalt, or carbon fibers; therefore, their contribution to load-bearing capacity may be more limited. Glass, basalt, steel, carbon, PVA, PE, and aramid fibers can provide different advantages depending on stiffness, bond strength, durability, and compatibility with the printable matrix [107,108,109,110,111,112,113]. The selection of fiber type should therefore be linked to the expected failure mechanism rather than treated as a simple strength-enhancing addition.

Engineered cementitious composites represent another relevant direction for fiber-reinforced printable materials. ECC-type mixtures reinforced with PVA, PE, steel, or hybrid fibers can exhibit strain-hardening and multiple cracking behavior when the matrix-fiber interaction is properly designed [109,110,111]. These features are attractive for 3DPC because they may improve ductility and crack control. However, ECC performance is highly sensitive to fiber dispersion, interfacial bond, matrix toughness, and rheological compatibility with the printing process. A mixture that performs well under casting conditions may not necessarily retain the same behavior after extrusion, because fiber alignment and layer interfaces alter the crack-bridging mechanism.

The use of high-performance fibers such as aramid fibers can improve crack resistance and mechanical performance, but cost, dispersion difficulty, and printability limitations must be considered [112,113]. Similarly, increasing fiber dosage may improve post-cracking behavior up to a certain level, but excessive fiber content can reduce flowability, increase extrusion pressure, cause blockage, disturb filament geometry, and weaken interlayer contact. In this respect, the optimum fiber content in 3DPC is governed by a compromise between mechanical reinforcement and printability.

Admixtures also affect the performance of fiber-reinforced printable mixtures. Viscosity-modifying admixtures can improve shape retention and reduce deformation after deposition, but excessive viscosity may impair pumping, reduce interlayer contact, and increase the risk of poor fiber dispersion [114]. Therefore, the design of fiber-reinforced 3DPC requires simultaneous control of fiber characteristics, rheology, extrusion stability, and interlayer bonding. A fiber system that improves strength in one direction but worsens printability or interlayer adhesion may ultimately intensify anisotropic behavior rather than mitigate it.

Overall, fiber reinforcement can reduce some consequences of anisotropy in 3DPC, but it does not automatically eliminate anisotropy. Its effectiveness depends on whether the fibers improve crack bridging across the weaker directions of the printed element, particularly the interlayer region. For structural applications, fiber reinforcement should therefore be integrated with toolpath design, interlayer control, and, where necessary, continuous or textile reinforcement systems.

A methodological limitation common to the fiber-reinforcement studies discussed above is that fiber orientation and dispersion are frequently assessed only indirectly, through resulting mechanical performance, rather than through direct quantification (e.g., X-ray computed tomography or image analysis of fiber distribution) [43,103,104,105,106,107,108,109,110,111,112,113]. This makes it difficult to separate the genuine effect of fiber bridging from confounding factors such as differences in matrix composition, curing regime, or specimen size between studies. In addition, comparisons of fiber type and dosage are often drawn across studies that used different printers, nozzle diameters, and extrusion rates, all of which are known to influence fiber alignment; this heterogeneity limits the extent to which optimum fiber dosages reported in one study can be generalized to other printing systems.

### 4.4. SEM Observations of 3DPC

Scanning electron microscopy is an important tool for examining the microstructural features that control the mechanical and durability performance of 3DPC. SEM observations can reveal pore morphology, hydration product distribution, fiber-matrix bonding, interfacial defects, microcracking, and damage patterns after mechanical or environmental exposure [115]. These observations are particularly valuable for printed concrete because the interlayer region, filament surface, and filament core may develop different microstructures during deposition and curing.

**Figure 8:**
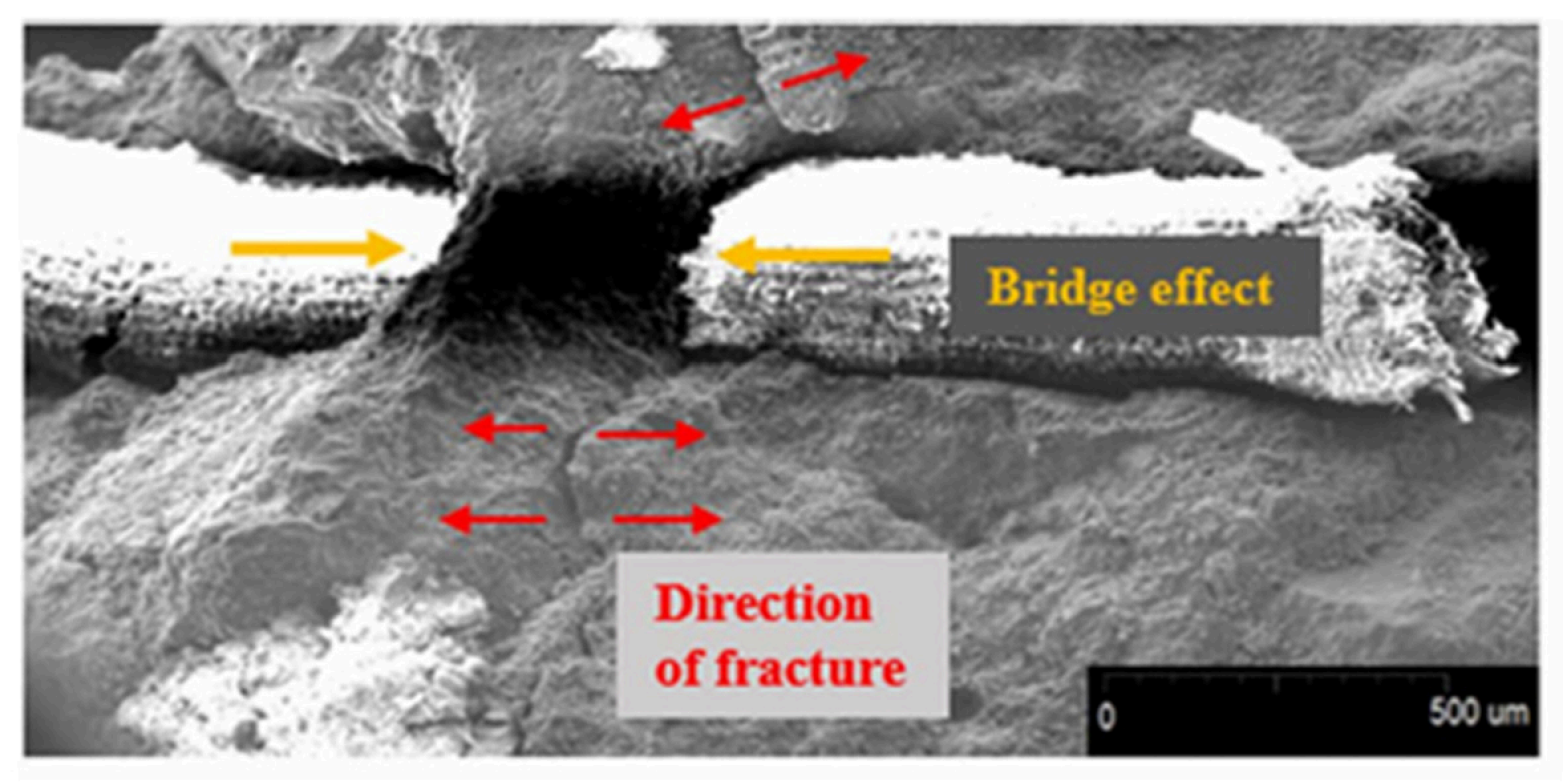
Fiber debonding and crack-bridging mechanism in fiber-reinforced 3DPC [116]. Licensed under CC BY 4.0.

In fiber-reinforced 3DPC, SEM analysis helps clarify the interaction between fibers and the cementitious matrix. During loading, fibers may contribute through crack bridging, pull-out resistance, and energy dissipation. However, weak fiber-matrix bonding may lead to debonding before the fiber can effectively transfer stress across a crack. Figure 8 illustrates this mechanism, where debonding and bridging occur around fibers during failure [116]. Such observations are important because the macroscopic benefit of fiber reinforcement depends strongly on the quality of the interfacial transition zone around the fiber. If the bond is weak, fibers may pull out prematurely; if the bond is excessively strong, brittle fiber rupture or matrix cracking may occur without sufficient energy dissipation.

**Figure 9:**
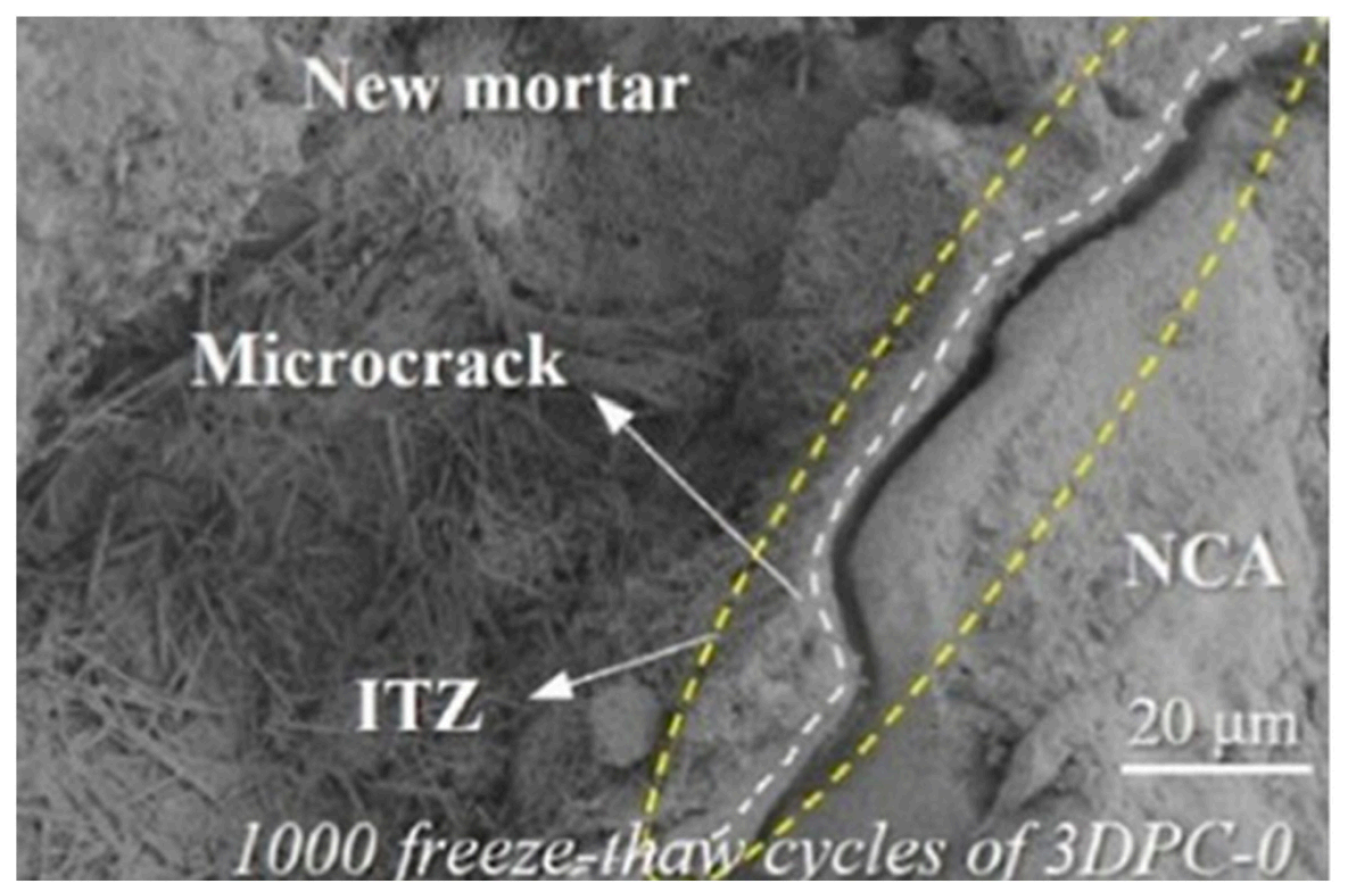
SEM image of a 3DPC sample after freeze–thaw damage [117]. Licensed under CC BY 4.0.

SEM observations are also useful for interpreting durability-related damage. After freeze–thaw exposure, the printed matrix may show microcracks, enlarged pores, disrupted hydration products, and weakened interfacial zones. Figure 9 shows the microstructure of a 3DPC sample after freeze–thaw damage, indicating that anisotropic pore networks and interfacial weaknesses can promote localized deterioration [117,118]. This type of damage is closely related to the pore architecture discussed earlier. If water accumulates along connected pores or interlayer defects, freezing can generate internal pressure and initiate cracks preferentially near the weak interface. Therefore, freeze–thaw deterioration in 3DPC is not only a durability problem; it is also linked to the anisotropic architecture of the printed material.

**Figure 10:**
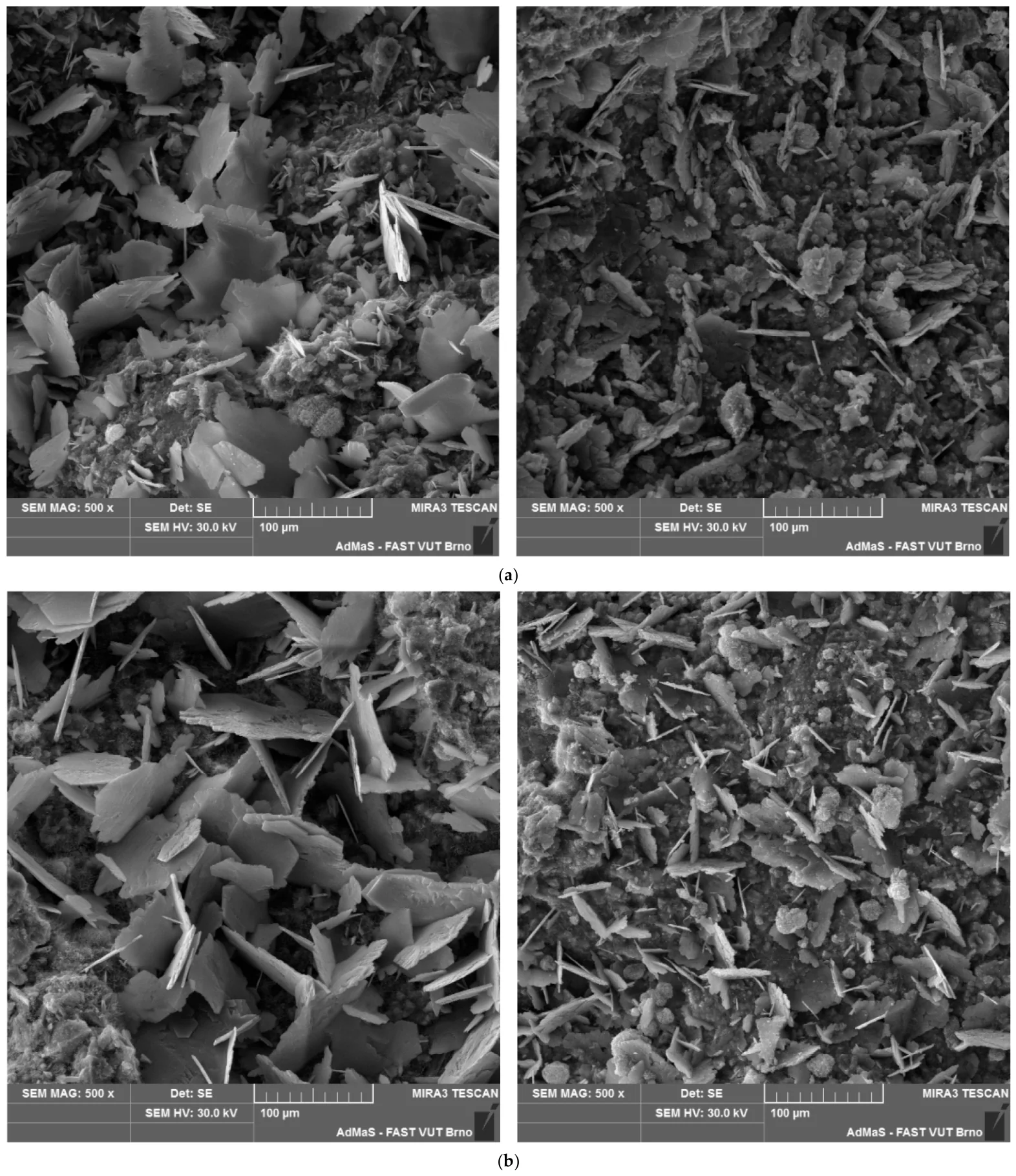
SEM images of the interlayer interface in a 3D-printed concrete mixture after 28 days of curing, showing the first (**left**) and second (**right**) layer at a 120 min delay time: (**a**) cast specimen; (**b**) printed specimen; adapted from [118]. Licensed under CC BY 4.0.

The distribution of hydration products is another key feature visible through SEM and EDS analysis. Calcium silicate hydrate, calcium hydroxide, ettringite, and other hydration products influence pore refinement, interfacial bonding, and mechanical strength. In printed concrete, the continuity of these products across the interlayer region is especially important. If the layer interface contains voids, insufficient contact, or locally disrupted hydration, the bond between layers may remain weaker than the filament core. Figure 10 presents SEM images of an OPC matrix before and after long-term immersion, with selected regions used for EDS analysis [118]. Such observations help identify changes in hydration products and microstructural integrity over time.

It should be emphasized that calcium hydroxide and C-S-H phases are not themselves “causes” of anisotropy. Rather, their distribution, continuity, morphology, and interaction with pores determine whether the microstructure can transfer stress effectively across the printed interface. A dense and continuous C-S-H network can improve bonding and reduce permeability, whereas discontinuous hydration products around pores or interlayer defects may reduce mechanical interlock and increase transport pathways. Therefore, the relevant issue is not the mere presence of hydration products, but their spatial development within the filament core and across the interlayer zone.

SEM evidence can also support the interpretation of mechanical interlocking. A well-bonded interface should show better continuity of hydration products, fewer connected pores, and stronger contact between layers. A weak interface, by contrast, may show flattened pores, microcracks, unhydrated regions, or discontinuities along the layer boundary. These features explain why printed specimens often fail along interlayer planes under flexural, tensile, or shear loading. When SEM observations are combined with mechanical testing, they provide a stronger explanation of anisotropic behavior than strength values alone.

Overall, SEM observations confirm that anisotropy in 3DPC originates from microstructural differences created during printing. Fiber-matrix debonding, interfacial pores, freeze–thaw microcracking, and discontinuous hydration products all contribute to the directional response of printed concrete. For this reason, SEM analysis should be used not only to describe microstructural damage, but also to connect printing parameters, interlayer bonding, fiber reinforcement, and durability performance.

## 5. Discussion

**Figure 11:**
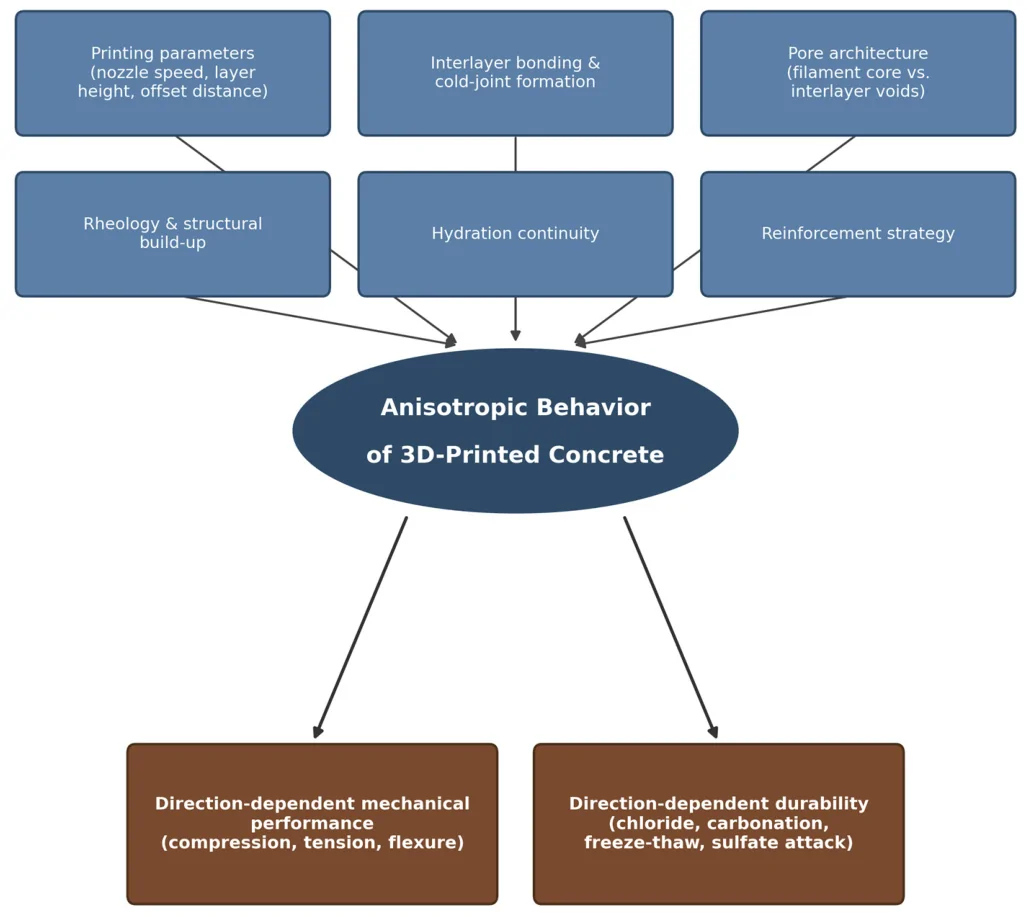
Synthesis of the general findings of this review: printing parameters, interlayer bonding and cold-joint formation, pore architecture, rheology and structural build-up, hydration continuity, and reinforcement strategy jointly govern the anisotropic behavior of 3D-printed concrete, which in turn determines direction-dependent mechanical performance and durability.

The anisotropic behavior of 3D-printed concrete is not governed by a single weakness, but by the material architecture generated during extrusion and layer-by-layer deposition. The reviewed studies indicate that the mechanical response of 3DPC is controlled by the interaction between printing direction, filament geometry, interlayer bonding, pore morphology, rheological evolution, hydration continuity, and reinforcement strategy [46,68,72,119,120]. Therefore, anisotropy should not be interpreted only as a difference between vertical and horizontal strength values. It should be understood as the structural expression of how the printed material is extruded, deposited, contacted, stiffened, hydrated, and loaded. Figure 11 summarizes these general findings, synthesizing how printing parameters, interlayer bonding, pore architecture, rheology, hydration continuity, and reinforcement strategy jointly govern the direction-dependent mechanical performance and durability of 3DPC.

One of the main points emerging from the literature is that the printed interface controls the transition from material behavior to structural behavior. In conventionally cast concrete, the material is usually placed as a continuous volume, and internal defects are governed mainly by mixture design, compaction, curing, and segregation resistance. In 3DPC, each deposited layer creates a new surface, and each surface may become a plane of weakness if the subsequent layer does not develop sufficient contact and bonding. This explains why the same printable mixture may exhibit different compressive, tensile, and flexural performance depending on the loading direction and the position of the interlayer region relative to the applied stress field [26,31,46,56,72,121]. When the applied stress crosses the layer boundary, interlayer adhesion, mechanical interlocking, pore distribution, and the continuity of hydration products govern the response. When the load is transferred mainly along the filament direction, the denser and more continuous filament core may dominate the response. This difference forms the basis of hierarchical anisotropy in 3DPC.

Pore architecture is central to this behavior. The absence of vibration or post-placement compaction allows voids to remain within the printed filament and, more critically, at the interface between layers. However, the effect of porosity in 3DPC cannot be evaluated only by total void content. The location, shape, orientation, and connectivity of pores are often more important than their volume alone. Pores concentrated at the interlayer region reduce the effective contact area, weaken stress transfer, and provide preferential paths for crack propagation [13,46,47,48,49,51]. Flattened or elongated pores along the printed interface are especially harmful because they increase local stress concentration and encourage fracture along the layer boundary. This is why pore morphology must be considered together with nozzle height, deposition pressure, offset distance, layer height, and interlayer time.

Cold joints represent the most severe form of interfacial discontinuity. Their formation is closely related to delayed deposition, surface drying, rapid structural build-up, and the loss of surface plasticity of the previously printed layer. When the next layer is deposited on a surface that is already too stiff, dry, or partially set, intimate contact between layers is reduced and the interface may develop as a porous and weak plane [31,52,55,56,57,122,123]. This weak plane affects not only strength but also the failure mode. Under flexural, tensile, or shear loading, cracks can initiate and propagate along the cold joint rather than crossing the filament body. Therefore, cold joints should not be treated as isolated construction defects. They are structural discontinuities that amplify anisotropy and may also accelerate durability degradation by increasing permeability.

Rheology is the link between printability and anisotropic performance. A mixture with rapid thixotropic build-up may provide better shape retention and buildability, but this benefit can be obtained at the cost of weaker interlayer bonding if the surface loses deformability before the next layer is placed. Conversely, a mixture with slower structural build-up may improve interfacial contact but may not support the weight of subsequent layers. This balance is central to 3DPC. The fresh material must be sufficiently stiff to maintain geometry, yet sufficiently plastic to merge with the previous layer. For this reason, anisotropy cannot be reduced simply by increasing mixture strength. It requires control of yield stress, plastic viscosity, thixotropic recovery, open time, layer interval, and surface condition [45,46,90,91,120,121].

Chemical development at the interface further explains why anisotropic weakness persists after hardening. The interlayer region may experience moisture loss, incomplete contact, and interrupted hydration before a continuous network of hydration products develops. If calcium silicate hydrate does not form continuously across the interface, the bond between layers remains weaker than the filament core. Connected pores and poorly hydrated regions can also facilitate the ingress of water, chloride ions, carbon dioxide, and sulfate ions [67,68,73,74,75,76]. This means that the mechanical and durability problems of 3DPC are closely connected. The same interlayer defects that reduce tensile or flexural strength may also increase carbonation depth, sulfate vulnerability, chloride ingress, and freeze–thaw damage. SEM- and EDS-based observations further show that the formation, morphology, and distribution of C-S-H are closely related to pore refinement, microstructural continuity, and mechanical performance [32].

Durability in 3DPC is therefore direction-dependent. The printed layer arrangement can create preferential transport paths, especially when pores and microcracks are aligned along the interlayer region. As a result, specimens extracted or exposed in different directions may show different resistance to water absorption, freeze–thaw cycles, carbonation, chloride penetration, and sulfate attack [68,77,80,81,83,122,123]. This behavior distinguishes 3DPC from ordinary cast concrete, where transport properties are generally less governed by deliberately layered material architecture. In printed concrete, surface texture, layer contours, interfacial porosity, and cold-joint formation can all contribute to more rapid ingress of aggressive agents. Hence, durability evaluation of 3DPC should consider the direction of exposure and the orientation of printed layers, not only the binder composition or compressive strength.

The lack of conventional reinforcement intensifies these problems. In reinforced cast concrete, steel bars, stirrups, meshes, or cages provide tensile continuity, crack control, ductility, and load redistribution. In 3DPC, integrating continuous reinforcement remains difficult because the reinforcement must be compatible with nozzle movement, layer deposition, cover control, anchorage, and bond development [14,15,90,93,94,95]. Unreinforced printed elements may perform satisfactorily in compression-dominated geometries, especially when the toolpath and global form are designed to follow the load path. Nevertheless, when bending, tension, shear, impact, thermal gradients, or long-term service loading govern the response, the absence of reinforcement crossing weak interfaces becomes a critical limitation. Geometry can reduce stress demand, but it cannot fully replace tensile continuity and crack control.

Fiber reinforcement offers an important but incomplete solution. Fibers can improve tensile resistance, flexural strength, crack-bridging capacity, and post-cracking behavior [71,103]. However, extrusion tends to orient fibers along the flow direction, which may improve the response along the filament axis while leaving the transverse and interlayer directions less effectively reinforced [60,103,104]. Therefore, the benefit of fibers depends on whether they bridge the weak interlayer region or merely strengthen the already favorable extrusion direction. Short fibers, polypropylene fibers, glass fibers, basalt fibers, steel fibers, PVA, PE, carbon, and aramid fibers each provide different mechanical and rheological effects, but none should be treated as a universal remedy for anisotropy [16,105,106,107,108,109,110,111,112,113,124,125,126].

Advanced reinforcement strategies such as FRP, textile reinforcement, and discrete minibar-type systems should also be interpreted through this framework. These systems can reduce anisotropic weakness when they provide tensile continuity across vulnerable planes and improve crack distribution [71,103]. However, they may introduce new challenges related to placement accuracy, bond quality, void formation around reinforcement, constructability, and compatibility with the printing process. A reinforcement strategy that increases nominal strength but disrupts layer continuity or creates additional defects may not improve structural reliability. Reinforcement design in 3DPC must therefore be coordinated with the printed architecture, expected stress field, and interlayer bonding conditions.

SEM observations support this interpretation by showing that anisotropy has a clear microstructural basis. Fiber pull-out, matrix cracking, interfacial voids, discontinuous hydration products, freeze–thaw microcracks, and weak fiber-matrix transition zones all help explain the macroscopic directional response of printed concrete [32,34,72,117,118,119]. SEM and EDS analyses are particularly useful because they reveal whether the interface is dense and continuous or porous and discontinuous. Strength values alone cannot explain why a specimen fails along the layer boundary. Microstructural evidence is needed to connect printing parameters, hydration development, pore architecture, and failure mode.

Taken together, the reviewed evidence indicates that anisotropy in 3DPC should be addressed through an integrated design approach. Mixture design must ensure pumpability, extrudability, shape retention, and adequate interlayer bonding. Printing parameters must control layer height, nozzle speed, deposition pressure, offset distance, and interlayer time. Toolpath design must consider load transfer and avoid placing weak interfaces in unfavorable stress directions. Reinforcement must be selected and positioned to bridge the weaker planes created by printing. Curing and environmental protection must preserve hydration continuity and reduce early moisture loss. Without this combined control, improving one property may worsen another; for example, increasing thixotropy may improve buildability but weaken interlayer adhesion, while increasing fiber content may improve crack resistance but reduce flowability and filament quality [36,37,46,50,90,91,92,116,121,122,123,126].

The main implication is that anisotropy is not an unavoidable defect of 3DPC, but it is also not a problem that can be solved by one parameter. It is a process-induced behavior that must be managed at several levels: material composition, rheology, filament formation, interface quality, toolpath geometry, reinforcement strategy, and curing.

It should be acknowledged that this review is itself constrained by the heterogeneity of the underlying literature: most cited investigations differ in printer hardware, mixture design, specimen scale, and testing protocol, which limits the extent to which quantitative results can be directly cross-compared or synthesized into unified design values. Rather than presenting a single set of governing thresholds, this review has therefore prioritized identifying convergent mechanisms and highlighting where methodological inconsistency currently prevents firmer, quantitative conclusions.

Future research should move beyond documenting directional strength differences and address several directions identified throughout this review. Standardized testing protocols are needed, particularly a common definition of interlayer time and a shared interlayer bond test geometry, since the methodological heterogeneity discussed in Section 3 and Section 4.1 currently limits cross-study comparison and the derivation of general design thresholds. In-situ characterization methods, such as X-ray computed tomography and digital image correlation, could be applied during printing rather than only afterward, allowing pore evolution and interlayer contact development to be characterized directly rather than inferred from post-mortem mechanical tests. Durability assessment would also benefit from longer timescales, extending beyond accelerated laboratory exposure to multi-year field exposure of full-scale printed elements, given that the representativeness of coupon-scale carbonation and freeze–thaw data for in-service performance remains unverified. Finally, reinforcement placement could be integrated into toolpath planning algorithms rather than treated as a post-hoc addition, allowing fiber orientation, FRP grid position, or textile placement to be optimized against the predicted stress field of a given geometry. Progress along these directions would support the translation of the mechanistic understanding synthesized in this review into structural design guidance for 3DPC.

## 6. Conclusions

This review examined the anisotropic behavior of 3D-printed concrete by linking the printed architecture, interlayer condition, pore structure, hydration development, durability response, and reinforcement limitations. The main conclusion is that anisotropy in 3DPC is not merely a directional difference in measured strength; it is a process-induced material characteristic created by extrusion, layer deposition, interfacial contact, and time-dependent evolution of the fresh cementitious matrix.

The reviewed literature indicates that the interlayer region is the governing zone for many mechanical and durability-related weaknesses of 3DPC. Weak contact between layers, insufficient mechanical interlocking, interfacial pores, cold joints, and discontinuous hydration products can reduce stress transfer and promote crack propagation along the printed interface. These features also provide preferential pathways for water, gases, and aggressive ions, thereby linking mechanical anisotropy with durability performance.

The role of geometry is important but not sufficient on its own. Toolpath design, filament arrangement, and structural form can reduce unfavorable stress concentrations and improve load transfer, especially in compression-dominated elements. However, geometric optimization cannot fully compensate for weak interlayer bonding, inadequate reinforcement, or poor microstructural continuity. For structural applications, geometry must therefore be coordinated with mixture design, rheological control, interlayer timing, curing, and reinforcement strategy.

Fiber, textile, FRP, and discrete reinforcement systems can reduce some consequences of anisotropy by improving crack control and tensile resistance. Nevertheless, their effectiveness depends on whether they bridge the weak interlayer regions and align with the expected stress field. Fiber addition alone should not be considered a complete solution, because extrusion-induced fiber orientation may strengthen the filament direction more effectively than the transverse or interlayer directions.

Overall, the reliable use of 3DPC requires anisotropy to be treated as a design parameter rather than as an unavoidable defect. Future studies should move beyond reporting directional strength differences and should combine mechanical testing with interfacial microstructural analysis, pore-network characterization, durability assessment, and reinforcement-layout evaluation. Such an approach is necessary for developing printed concrete elements with predictable structural performance and long-term durability.

## Figures and Tables

**Table 1:** Scope of recent reviews on the anisotropic behavior of 3D-printed concrete relative to the present study.

Review	Primary Scope	Interlayer Bonding	Pore Architecture & Durability	Reinforcement & Numerical Modeling
Ding, Xiao & Mechtcherine (2023) [33]	Critical synthesis of interlayer region microstructure and mechanical properties	Central focus of the review	Discussed only in relation to interlayer microstructure; durability mechanisms not addressed	Not addressed
Baktheer & Classen (2024) [34]	Numerical modeling strategies for the anisotropic behavior of hardened 3DPC	Addressed indirectly through interface-based constitutive models	Not addressed	Central focus: phenomenological, interface-based, and discrete modeling categories
Present study	Unified architectural, physical, and chemical basis of anisotropic behavior	Addressed as part of the physical mechanism category, linked to cold-joint formation and mechanical interlocking	Addressed in detail, including pore location, morphology, and durability-related transport mechanisms	Reinforcement addressed (fiber, textile, FRP, discrete systems); numerical modeling referenced but not synthesized in depth

**Table 2:** Conceptual framework linking the 3D printing process to the structural and durability consequences of anisotropy, and the corresponding sections of this review.

Framework Stage	Governing Mechanism	Key Factors	Covered In
Process	Extrusion and layer-by-layer deposition	Nozzle geometry, deposition pressure, interlayer time, rheology	Section 2.1 and Section 4.1
Architecture	Interlayer interface and pore architecture	Bond quality, cold-joint formation, pore morphology and connectivity	Section 2.2 and Section 2.3
Mechanical response	Direction-dependent strength and failure mode	Loading direction relative to interlayer plane, mechanical interlocking	Section 2.1, Section 4.1 and Section 5
Durability response	Direction-dependent transport and degradation	Carbonation, chloride ingress, sulfate attack, freeze–thaw, hydration continuity	Section 3
Mitigation	Reinforcement strategies	Fiber, textile, FRP, discrete reinforcement; bridging weak planes vs. reinforcing filament direction	Section 4.2, Section 4.3 and Section 4.4
Synthesis	Integration of all mechanisms	Combined design approach across mixture, process, and reinforcement	Section 5 (Figure 11)

**Table 3:** Anisotropy ratios reported across studies as a function of mixture, printing geometry, interlayer time gap, and test configuration.

Source	Mixture/Material	Layer Height	Nozzle/Deposition Speed	Interlayer Time Gap/Test Configuration	Reported Result	Anisotropy Ratio (Quantitative)
Xiao, Liu & Ding [21]	Cement mortar, fc = 31 MPa	Not stated as “layer height”; filament (specimen) height was 10 mm for compression and 15 mm for flexural specimens	Not reported	Filament 20 × 10 mm (compression), 30 × 15 mm (flexure); no interlayer time gap	Compression: Fc > Fx > Fz > Fy. Flexure: Fz ≈ Fc ≈ Fy > Fx (source reports these as bar-chart results only; no numerical strength table is given for its own specimens)	Not quantifiable—the source does not tabulate absolute strength values for its own specimens (bar charts only), so a precise ratio cannot be computed
Liu et al. [43]	Sand:cement = 1.5; 0.6% polypropylene fiber (confirmed in source)	12–14 mm (reported directly in the source as the printing-belt/filament height)	Not reported	Filament width 23–26 mm; no interlayer time gap	Compression: DX = 35.66 MPa (highest); DY = 87.07% of DX; DZ = 82.56% of DX	DY/DX ≈ 0.87; DZ/DX ≈ 0.83
Aminpour & Memari [20]	Normal-weight and lightweight (40% EPS) printable concrete	Not reported	Not reported	30–35 s (minimal) vs. 5 min interlayer time gap	Compressive strength reduced 4.5–53% (NWC) and 11–49% (LWC) by printing and delay	Anisotropic coefficient (source’s own term/metric) multiplied by delay ×6.98 (compressive), ×7.81 (flexural), ×10.81 (shear) for NWC; ×3.95, ×5.0, ×4.2 respectively for LWC
Maroszek et al. [44]	Portland cement-based mineral composite	Not stated directly; inferable from specimen preparation—flexural specimens (40 × 40 × 160 mm) were cut to include four printed layers, implying a layer height of ≈10 mm	Not given for the tested specimens; the source separately reports maximum achievable printing speeds of 100 mm/s (20 mm nozzle) and 50 mm/s (40 mm nozzle) in the context of justifying its time-gap values, not as the speed actually used for these specimens	0, 25, and 50 min interlayer time gap	Flexural strength (perpendicular) fell from ≈4.8 MPa (0 min) to ≈3.5 MPa (50 min), a ≈27% reduction; flexural strength parallel to the layers was ≈60% lower than perpendicular, regardless of gap; compressive strength fell by ≈17% (perpendicular) and ≈10% (parallel) at 50 min	Flexural, parallel/perpendicular ≈ 0.40 (confirmed: source states parallel values are ~60% lower). Flexural, 50 min/0 min (perpendicular) ≈ 3.5/4.8 ≈ 0.73

**Table 4:** Porosity of 3D-printed cementitious materials differentiated by filament core (intralayer) and interlayer region, as measured by X-ray computed tomography and BSE-SEM.

Source	Material/CT Method	Region Investigated	Reported Porosity	Remarks
Kruger et al. [64]	3DPC mortar; X-ray µCT, 15 µm voxel resolution	Cast reference (no pumping or vibration)	6.8% (COV 8.9%)	Baseline for comparison with printed specimens
Kruger et al. [64]	3DPC mortar; X-ray µCT, 15 µm voxel resolution	Filament core, single printed layer	7.9% (COV 7.9%)	Printed filament core porosity slightly higher than the cast reference
Kruger et al. [64]	Full filament segment (40 × 40 × 10 mm); X-ray µCT, 22.5 µm voxel resolution	Filament core (larger sampling volume)	4.2% (COV 30.9%)	Larger voxel size cannot resolve the smallest pores detected in the molded specimens; voids elongated and tri-axial ellipsoidal, oriented along the print direction
Kruger et al. [64]	3DPC mortar; X-ray µCT, 15 µm voxel resolution	Vertical interlayer (0 min pass time)	8.0%	Comparable to the horizontal interlayer porosity below
Kruger et al. [64]	3DPC mortar; X-ray µCT, 15 µm voxel resolution	Horizontal interlayer, pass time 0–60 min	7.7% (0 min), rising to a local peak of ≈14% (60 min)	At short pass times, interlayer porosity did not differ significantly from the filament core (≈8%); longer pass times produced larger, elongated interlayer voids
Van Der Putten et al. [61]	3DPC mortar; BSE-SEM phase analysis	Bottom/top layer, 0 min interlayer time	Bottom 7.6%; top 8.4%	Comparable unhydrated cement (UH) content in both layers, confirming negligible moisture exchange at the interface
Van Der Putten et al. [61]	3DPC mortar; BSE-SEM phase analysis	Bottom/top layer, 10 min interlayer time	Bottom 10.8%; top 11.4%	Highest capillary porosity and air-void content (3.4%, RapidAir) recorded at this interval, attributed to peak moisture exchange between layers
Van Der Putten et al. [61]	3DPC mortar; BSE-SEM phase analysis	Bottom/top layer, 30 min interlayer time	Bottom 4.9%; top 8.4%	Porosity and unhydrated cement content decreasing relative to the 10 min interval
Van Der Putten et al. [61]	3DPC mortar; BSE-SEM phase analysis	Bottom/top layer, 60 min interlayer time	Bottom 1.2%; top 8.5%	Air-void content (2.8%) remained above the 0 min baseline despite lower bottom-layer capillary porosity

**Table 5:** Interlayer time-gap effects on bond strength and related mechanical/durability properties, reported across studies.

Source	Material/Test Method	Interlayer Time Gaps Investigated	Reported Result	Measurement Type
Tay et al. [66]	Cement paste, direct tensile bond test (ASTM C1583)	1, 5, 10, and 20 min	Bond strength decreased logarithmically from ≈0.82 MPa (1 min) to ≈0.16 MPa (20 min); the reduction was most pronounced between 1 and 5 min and became insignificant beyond 10 min	Direct interlayer bond strength
Xu et al. [67]	3DPC mortar, oblique shear bond test, 28-day curing	0 (T0), 1, 2, 3, 5, 10, 20, 30, and 60 min	Shear bond strength dropped from 14.41 MPa (T1) to 8.04 MPa (T10, −44.2%) and to 7.11 MPa (T60, ≈half of T1); the first 10 min were most critical, with a comparatively stable trend thereafter	Direct interlayer bond strength (shear)
Van Der Putten et al. [68]	Four-layered printed mortar, chloride diffusion and colorimetric penetration analysis	0 min (T0) vs. 30 min (T30)	Bond strength not measured directly; T30 showed markedly higher interlayer porosity and a shift from equidistant to interlayer-dominated chloride and CO_2_ penetration, indicating progressive weakening of the interface with increasing time gap	Indirect—porosity and transport (durability) consequence of the time gap, not a direct bond-strength measurement
Maroszek et al. [44]	Portland cement-based mineral composite; flexural, compressive, direct tensile, and splitting tensile tests	0, 25, and 50 min	Flexural strength (perpendicular) fell from ≈4.8 to ≈3.5 MPa (−27%) at 50 min; compressive strength (perpendicular) fell by ≈17% at 50 min, ≈10% parallel; the direction parallel to the layers was consistently less sensitive to the time gap	Indirect—bulk flexural/tensile/compressive strength of printed specimens, not a dedicated interlayer bond test

## Data Availability

No new data were created or analyzed in this study. Data sharing is not applicable to this article.
